# A structure-based computational workflow to predict liability and binding modes of small molecules to hERG

**DOI:** 10.1038/s41598-020-72889-5

**Published:** 2020-10-01

**Authors:** Subha Kalyaanamoorthy, Shawn M. Lamothe, Xiaoqing Hou, Tae Chul Moon, Harley T. Kurata, Michael Houghton, Khaled H. Barakat

**Affiliations:** 1 Faculty of Pharmacy and Pharmaceutical Sciences, University of Alberta, Edmonton, AB Canada; 2 Department of Pharmacology, Faculty of Medicine and Dentistry, University of Alberta, Edmonton, AB Canada; 3 Department of Medical Microbiology and Immunology, Faculty of Medicine and Dentistry, University of Alberta, Edmonton, AB Canada; 4 Li Ka Shing Applied Virology Institute, University of Alberta, Edmonton, AB Canada; 5 Department of Chemistry, University of Waterloo, Waterloo, ON Canada

**Keywords:** Ion channels, Molecular medicine

## Abstract

Off-target interactions of drugs with the human ether-à-go-go related gene 1 (hERG1) channel have been associated with severe cardiotoxic conditions leading to the withdrawal of many drugs from the market over the last decades. Consequently, predicting drug-induced hERG-liability is now a prerequisite in any drug discovery campaign. Understanding the atomic level interactions of drug with the channel is essential to guide the efficient development of safe drugs. Here we utilize the recent cryo-EM structure of the hERG channel and describe an integrated computational workflow to characterize different drug-hERG interactions. The workflow employs various structure-based approaches and provides qualitative and quantitative insights into drug binding to hERG. Our protocol accurately differentiated the strong blockers from weak and revealed three potential anchoring sites in hERG. Drugs engaging in all these sites tend to have high affinity towards hERG. Our results were cross-validated using a fluorescence polarization kit binding assay and with electrophysiology measurements on the wild-type (WT-hERG) and on the two hERG mutants (Y652A-hERG and F656A-hERG), using the patch clamp technique on HEK293 cells. Finally, our analyses show that drugs binding to hERG disrupt and hijack certain native—structural networks in the channel, thereby, gaining more affinity towards hERG.

## Introduction

Drug safety is one of the primary factors that determine the fate of any drug discovery and development program. Almost 90% of compounds that enter clinical trials fail to meet the safety, efficacy and pharmacokinetic/pharmacodynamic requirements to obtain the Food and Drug Administration approval^1,2^. Such failures due to drug safety lead to high attrition rates and also affect patients’ health. There were a number of instances in the past when the marketed drugs with side effects caused severe implications, for instance, thalidomide (an antiemetic agent) that was administered to pregnant women resulted in severe birth deformities in the new borns^3,4^. Therefore, continuous efforts are being made to detect, monitor, and prevent adverse side effects from pharmaceutical products^5^.

Cardiotoxicity remains one of the most serious side effects caused by the off-target interactions of drugs with cardiac ion channels important for cardiac rhythmicity and contractility. Amongst various ion channels in the heart, the human ether-à-go-go-related gene (hERG) channel is an important off-target for drugs that induce cardiotoxicity. hERG is a potassium-selective ion channel that plays a crucial role in cardiac repolarization by conducting the rapidly activating delayed rectifier current (I_Kr_)^6^. Blockade of the hERG channel results in the prolongation of the repolarization phase of the cardiac action potential, a condition called long QT syndrome (LQTS). Such delayed repolarization introduces the risk of cardiac arrhythmias, Torsades de pointes (TdP) and sudden cardiac death. Several drugs have been withdrawn from the market as they induced cardiac arrhythmia through off-target interactions with hERG, such as terfenadine (an antihistamine drug), cisapride (a serotonergic agent), grepafloxacin (an antibacterial agent), and droperidol (an antidopaminergic drug)^7–9^. Therefore, the hERG liability of compounds is monitored very early in the drug discovery process.

Although a number of high throughput assays and electrophysiology measurements are available to test small molecules binding to hERG^10^, the cost of these experiments remain to be a drawback in using these methods^9^. To augment that, in silico techniques, especially machine learning-based classifiers, have been developed to provide a reasonably accurate prediction of hERG liability at low cost^9,11–14^. These classifiers have been trained using librairies of diverse structures of known hERG blockers and can screen a large number of compounds against hERG very rapidly. Hence, currently available technologies can identify hERG liable compounds efficiently enough. However, there is still an important detail to this liability that needs to be addressed. That is, understanding the mode of binding of hERG blockers. An access to these interactions can be extremely useful in understanding the reasons behind hERG liability. In silico structure-based methods were sought to help in this regard^15–18^. Until recently, homology models of the hERG channel^15–23^ have been the only source to gain insights into these mechanisms. Although all earlier models agree that hERG blockers bind predominantly at the large central cavity of the channel, there is no clear agreement on their predicted binding modes^8,24^. The binding mode hypotheses varied depending on the template and on the methods used. In this context, the recently reported cryo-EM structure of the hERG channel^25^ (PDB ID: 5VA1) provides a wealth of structural information about the channel and also makes it possible to generate a more reliable hypothesis.

**Figure 1:**
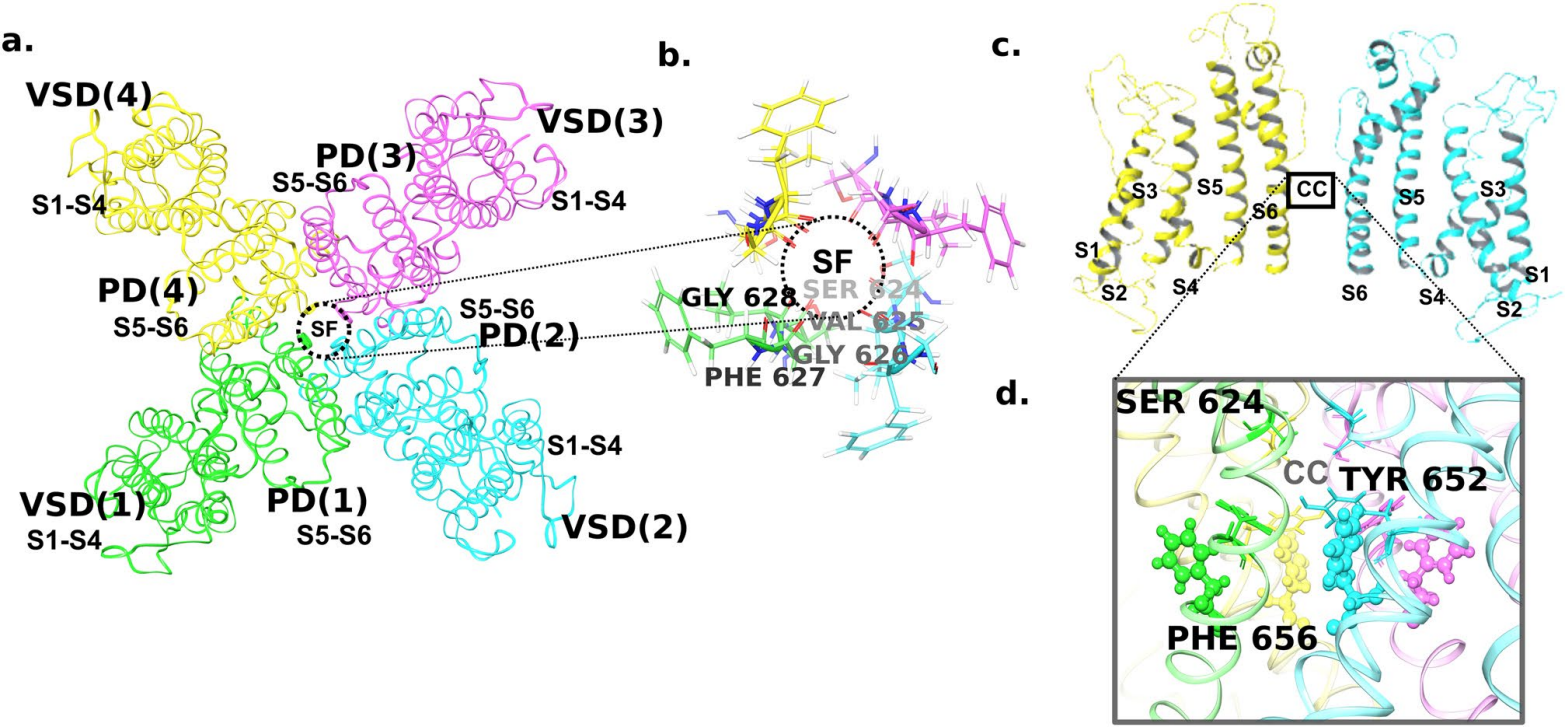
hERG channel (**a**) Extracellular view of the cryo-em resolved structure of the hERG channel (PDB ID: 5VA1); *VSD* Voltage Sensing Domain, *PD* Pore Domain, *SF* selectivity filter. Zoomed in view of the channel pore composed of the residues in the SF is shown (**b**) Rotated structure of the channel with the extracellular side at the top. Only two subunits are shown for clarity. The helices are numbered from S1-S6; S4-S5L is the linker connecting the VSD and PD of the subunits; CC refers to the central cavity; PH refers to the pore helix (**c**) Zoomed in view of the central cavity with the two key aromatic residues PHE656 (shown in ball and stick) and TYR652 along with the SF residue, SER624 are shown in sticks.

The hERG1a channel presents as a quartenary structure that is composed of four polypeptides (or subunits) of 1159 residues. The four subunits co-assemble to form a functional channel with a central pore through which the K^+^ ions permeate (Fig. 1a). Each polypeptide is divided into an N-terminal PAS domain, a transmembrane domain-formed by a voltage sensing domain (VSD), a pore domain (PD), and a C-terminal cyclic nucleotide-binding homology domain (CNBHD). The VSD of each monomer has four continuous transmembrane helices (S1–S4) that play a crucial role in sensing the membrane potential of the channel (Fig. 1b). The pore domain corresponds to the segments from S5 to S6 helices, which include S5 helix, S5-pore helix linker, pore helix, selectivity filter (SF) and the S6 helix (Fig. 1b). The carbonyl oxygen atoms of the residues ‘SVGFG’ in the selectivity filter provide a suitable electrostatic environment for selective transport of K^+^ ions (Fig. 1a). Mutational analyses identified the cavity (central cavity, cc) beneath the selectivity filter, formed by the four subunits, as the major drug binding site in hERG^26,27^. Especially, PHE656 and TYR652 were identified as key residues for hERG inhibition^8,16^ (Fig. 1c). The four copies of these two key residues (*i.e.,* two from each of the four subunits) leads to an unusual promiscuity to bind a variety of drug moieties and imposes a serious challenge in identifying the mode of binding of drugs in the hERG central cavity. Acquiring knowledge on the mode of binding of drugs would help in the development and/or re-design of the potential drug candidates. Although successful optimizations of drug candidates to reduce their hERG liability have been performed through synthetic medicinal chemistry routes^8,28^, it is not always feasible to achieve this and the medicinal chemistry efforts to reduce hERG affinity are not trivial^29^. Since a lead optimization process often involves compounds with a narrow range of hERG inhibition from 1–30 μM^30^ to build the structure–activity relationship, it is necessary to have low-cost, high-precision methods that can identify the compounds (or analogs) falling in the lower and upper extremities of this range. Therefore, in silico structure-based methods that provide access to atomistic details of the drug-hERG interactions and efficiently rank the analogs by their affinity could be highly useful in selecting the appropriate functional groups for optimization.

**Figure 2:**
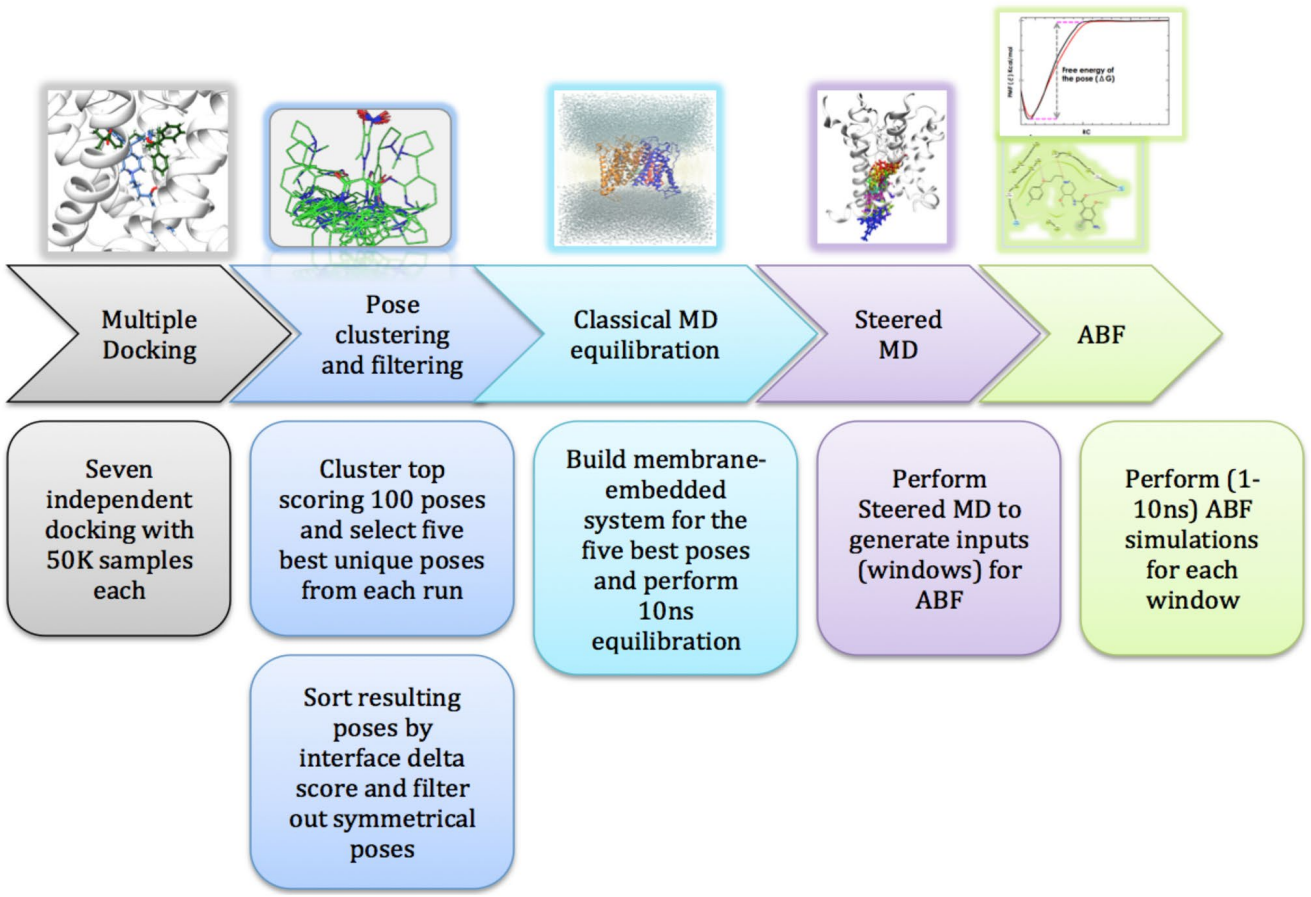
Insilico workflow employed in this study for predicting the relative ranking of structurally similar small molecule analogs.

In this work, we employed a sophisticated in silico workflow (Fig. 2) to study small molecule analogs that bind the hERG channel. The workflow incorporates structure-based methods, such as molecular docking, molecular dynamics-based binding free energy calculations, and potential of mean force (PMF) calculations using the Adaptive Biasing Force (ABF) simulation. We used two classic hERG binders as examples, namely, Cisapride and Ranolazine and included three additional analogs for each of these two compounds, which were previously derived using medicinal chemistry optimization efforts (Table 1). Cisapride is a gastroprokinetic drug that acts as an agonist to serotonin 5-hydroxytryptamine receptor 4 (5HT-4) with a binding affinity of ~ 14 nM^31,32^. Cisapride was withdrawn from the market due to its unwanted side effects including LQTS, TdP and sudden death. It was identified to inhibit the hERG1 channel with an affinity of ~ 9 nM^32^. On the other hand, mosapride and two other analogs (13a and 17a) of cisapride from literature^33^, was shown to have a lower inhibitory potencies towards the hERG channel with ~ 4 μM^32,34^ and > 10 μM^33^, respectively. Similarly, ranolazine, an anti-anginal drug and a sodium channel blocker that has an IC_50_ value of ~ 8 μM^35^ to the hERG channel and its’ molecular analogs lidocaine, pilsicainide, and mepivacaine that has very weak inhibition to hERG with an IC_50_ of ~ 141 μM^35^, ~ 20 μM^36^ and 156 μM^37^, respectively were chosen.

**Table 1:** Drugs selected in this study with their reported IC_50s_ from literature.

Compound	2D Structure	hERG IC_50_ from literature (M)	Experimental technique	References
Cisapride		9.4 × 10^–9^	Patch clamp HEK293 cells @ 23 ± 1 °C	Toga et al.
Mosapride		4.8 × 10^–6^	Patch clamp HEK293 cells @ 23 ± 1 °C	Toga et al.
13a		> 10 × 10^–6^	hERG fluorescence polarization assay	Park et al.
17a		> 10 × 10^–6^	hERG fluorescence polarization assay	Park et al.
Ranolazine		8.03 × 10^–6^	Patch clamp HEK293 cells @ 37 ± 1 °C	Du et al.
Lidocaine		141.77 × 10^–6^	Patch clamp HEK293 cells @ 37 ± 1 °C	Du et al.
Pilsicainide		20.4 × 10^–6^	Patch clamp HEK293 cells @ 34 ± 1 °C	Wu et al.
Mepivacaine		156.2 × 10^–6^	Patch clamp HEK293 cells @ 34 ± 1 °C	Wu et al.

The current selection of molecules include one high affinity blocker with a nanomolar range inhibition (cisapride), three mid-affinity blockers (mosapride, ranolazine, and pilsicainide) with < 30 μM inhibition, two low-affinity blockers (lidocaine and mepivacaine) with > 100 μM inhibition and two mid/low affinity blockers, 13a and 17a, with > 10 μM inhibition. It is important to note that the above-mentioned inhibitory potencies are not direct indicative of the hERG blocking potencies of these molecules, as the significance of the hERG blockade induced by small molecules is always assessed in congruence with the actual target binding affinity. For example, ranalozine was estimated to have ~ 8 μM^35^ inhibition constant to the hERG channel, however, it is considered as one of the potential hERG blockers due to its micromolar range therapeutic concentrations (towards the sodium channel)^38^. While, the same in silico workflow can be adapted to check for the actual target affinity, we have focused here only on the affinity to the hERG channel and therefore, the above classification correspond only to the hERG channel affinity.

By using two classes of structurally similar small molecules, we show that the proposed workflow with the MD-based methods has better sensitivity in differentiating these analogs when compared to the conventional docking-based classification. Further, we discuss the low-energy states or the likely-mode of binding of the ligand predicted from the ABF simulations for the selected compounds. We also confirmed the binding and key residue interactions by performing Polarization binding assay and electrophysiology measurements on the wild type (WT) and mutant (Y652A and F656A) hERG channels. The proposed computational workflow can therefore be useful for classifying the compounds by the hERG binding affinity and the molecular insights obtained from the analyses can aid in medicinal chemistry optimization for reducing the hERG liability.

## Results and discussion

### Docking energy-based ranking

**Figure 3:**
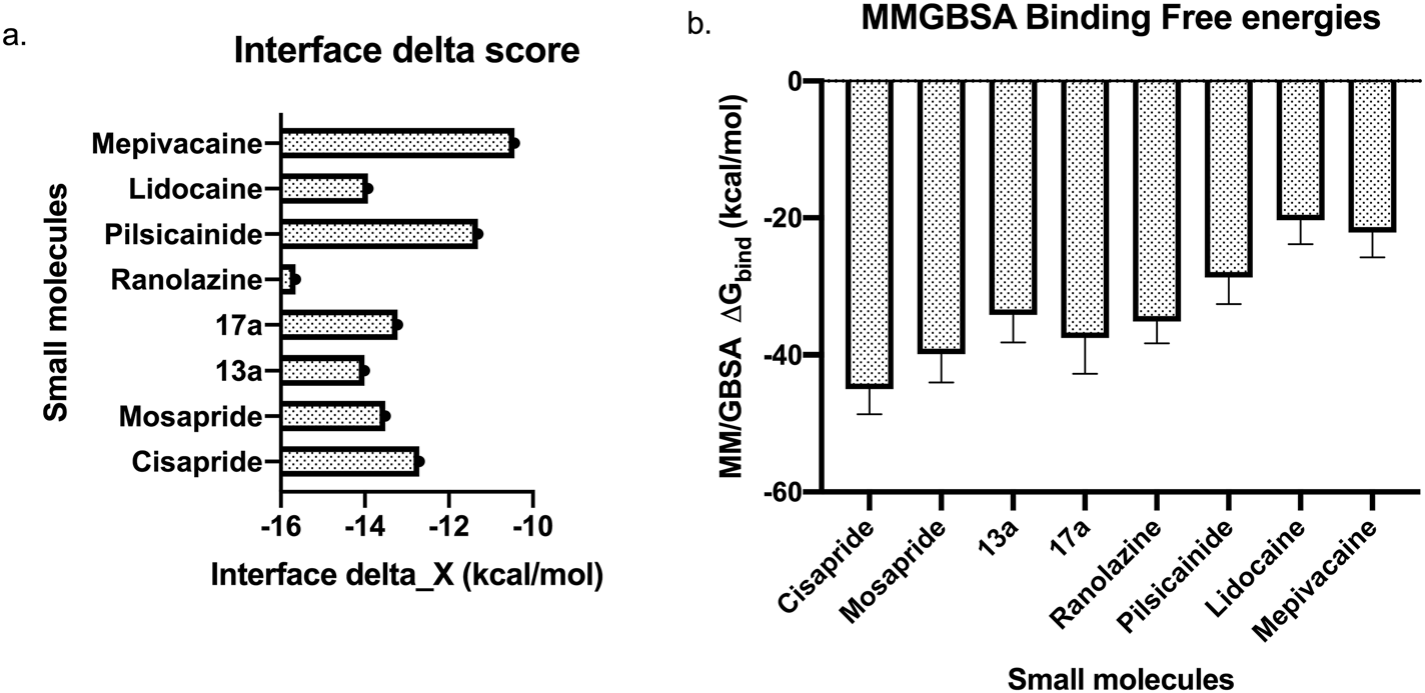
Distribution of the (**a**) docking scores, and (**b**) Post-MD binding free energies estimated using the MMGBSA method on snapshots sampled from the MD trajectories. Error bars represent standard deviation.

All tested compounds were individually docked into the central cavity of the hERG channel, and the resultant drug-channel complexes were clustered and filtered to remove redundant and symmetrical poses. For each compound, the top five poses based on the estimated binding energies (i.e., interface delta_X scores from Rosetta) were selected (please refer to the methods section for details) and analysed. The selected poses had at least 2 Å RMSD between each other (supplementary Table 1 and supplementary Figs. 1–4). The distribution of the binding energies of the best-ranked pose for each drug-channel complex are illustrated in Fig. 3a. As shown in this figure, the binding energies of the compounds are distributed along a 6 kcal/mol window (− 16 to − 10 kcal/mol). While the known low-affinity molecules, such as mepivacaine and pilsicainide clearly showed low interface delta energies, the energies of some other molecules did not agree to the expected affinity-based ranking. For instance, the interface delta energy of ranolazine (a micromolar affinity molecule) was better than cisapride (a nanomolar affinity molecule), which contradicts the reported experimental affinities^31,32,35^ of these molecules . Similarly, the other analogs of cisapride, 13a and 17a, showed better interface delta scores than the parent molecule.

Five best-scoring poses that differed in their positions, orientations, and the number and types of interactions with the key residues present in the central cavity (supplementary Fig. 1–4) were selected for further analysis. All of the chosen drugs exhibited parallel mode of binding^8,24^ (i.e., drugs bind parallel to the pore axes), in one or more poses. The perpendicular mode of binding^8,24^ (in which drugs bind perpendicular to pore axes) was observed only in smaller molecules, such as lidocaine, pilsicainide, and mepivacaine, while the longer molecules exhibited an inverted ‘U’ shaped or an inverted ‘L’ shaped (semi-perpendicular) binding modes. All the drugs, irrespective of their affinity, showed potential interactions with the TYR652 residue. For instance, the docked poses showed the possibility for cisapride and its’ analogs to interact with one to all four TYR652 residue. However, none of the best-scoring poses for cisapride, 13a, and 17a showed a π- or hydrogen bond interaction with PHE656 residue. On the other hand, one of the best pose identified for mosapride showed a hydrogen bond to the backbone of a PHE656 residue. Similarly, ranolazine, lidocaine, pilsicainide, and mepivacaine poses were found to interact with one to three TYR652 residue and none of them were seen to have a π- or hydrogen bond interaction with PHE656 residue.

It is well known that PHE656 residue plays a key role in drug-induced blockade of the hERG channel. Given its’ significance, the orientation of PHE656 residue observed in the cryo-EM structure probably needs a reorientation to facilitate high-affinity block of some of these compounds^16^. Although, we have employed a flexible docking protocol to account for receptor flexibility, the lack of interactions with PHE656 in almost all the best-scoring poses illustrates the need for an extended sampling of the conformational space around the central cavity region. Further, the difference in the interface delta energies for most of the poses were trivial, therefore, we decided to use multiple docking poses for our subsequent molecular dynamics (MD) simulations. We believe that employing MD simulations would help in resolving two prime issues of docking^39–42^, which includes, accounting for all-atom protein and ligand flexibility, and including the effects of explicit waters and lipid membrane, and also improve the accuracy of the predictions.

### MD and binding free energy-based ranking

We chose to consider multiple possible docking solutions, therefore, for each compound, we selected the top five poses from docking simulations to move forward for MD-based equilibration and free energy-based re-scoring. To model these complexes in a physiologically relevant environment, each drug-hERG complex was embedded in a lipid bilayer construct and was equilibrated with explicit water and ions (refer to methods) for 10 ns of simulation time. The RMSDs calculated for each drug-bound complex system (supplementary Fig. 5) stabilized mostly at around 4 Å. Later, the molecular mechanics—generalized Born surface area method (MM-GBSA) was used to estimate the binding free energies for each pose (supplementary Table 2) using twenty-one snapshots that were sampled at equal intervals from the respective equilibration trajectories. The best binding free energy (i.e., the lowest negative energy) for each compound was selected (Fig. 3b) and the overall relative ranking of all compounds in this study was assessed.

Our data show that the MM-GBSA rescoring predicted the relative order of the drugs accurately. For example, cisapride, the strongest hERG blocker amongst all compounds in this study, was predicted with the lowest (most negative) binding free energy score of ~ − 45 kcal/mol. In the case of the weakest blockers, Lidocaine (~ 141 μM) and Mepivacaine (156 μM), their binding free energies were predicted as ~ − 26 kcal/mol and − 22 kcal/mol, respectively, which were much weaker than the binding free energies of other compounds. However, the MM-GBSA rescoring struggled when comparing the compounds that showed mid-range and closer affinities. For example, the reported hERG channel affinities^31,32,35–37^ of mosapride, 17a and ranolazine were ~ 4 μM, > 10 μM, and 8 μM, respectively. Their MM-GBSA scores showed an energy difference of ~ 2 kcal/mol, whereas, the standard deviation of the estimates was over ± 3 kcal/mol thereby placing them in almost same footing. Further, based on the binding free energies predicted for 17a (− 37.57 kcal/mol), this molecule can be classified as the one with high-/mid-affinity to the hERG channel, however the literature data suggests that this could be a mid to low-affinity range (> 10 μM) hERG blocker. Nevertheless, it is clear that enhancement of plasticity in the drug-hERG complex during MD simulations was helpful in improving the relative ranking of the different compounds chosen in this study.

The discrepancies and the overlapping energies between some of the compounds can be attributed to the nature of the MMGBSA method being an end-point approach, In the end-point approach, the binding free energies are estimated based on the final ligand–protein complex and the intermediate steps leading to this binding reaction are not taken into account^43,44^. In order to improve the precision of the predictions, we employed a PMF-based method^45^ using ABF simulations and estimated the free energies by sampling different intermediate steps along the (un)binding process. Further, the ABF method provides enhanced sampling of conformations, and therefore can be expected to find more accurate low-energy states of the bound complex for the ligand in the protein environment (or in other words, a likely favourable mode of binding).

### Adaptive biasing force (ABF) and PMF-based ranking

ABF maps the free energy landscape of a protein-drug (un)binding through consecutive geometrical transformations. In the current study, we employed ABF to construct the energy landscape of the “unbinding” reaction for each compound by removing it gradually from their initial binding locations as suggested by docking. During these unbinding processes, a continuously adapted biasing force was added to the equations of motion to produce a flat free-energy surface for enhanced sampling. In the long time scale, a Hamiltonian with no average force acting along the reaction coordinate is produced and the free energies of the ligand was estimated from the PMF profiles^46^, The minimum along the reaction coordinate in the PMF is expected to correlate with the location of the favourable and likely binding mode within the hERG channel.

All top five poses, for each ligand, from docking were included in this calculation. During the ABF simulation, the direction through the intracellular gate was selected as the reaction coordinate (RC) in order to study the change of free energy as the ligand moves (unbinds) from the binding site (i.e., pore of the channel) and leaves the channel along the RC. The total length of the RC was split into multiple small windows of ~ 2 Å and the ABF simulations were performed in each window and the PMF required to exit the channel were constructed. If a ligand was to find a low energy point than the initial position, then the free energy drops; otherwise, the free energy value rapidly increases to only reach a plateau upon the ligand’s complete exit from the channel. Thus, ABF and PMF profiles can provide both a quantitative measure for distinguishing the drugs based on their affinity to the target; and qualitative insights about the binding mode of drugs.

**Figure 4:**
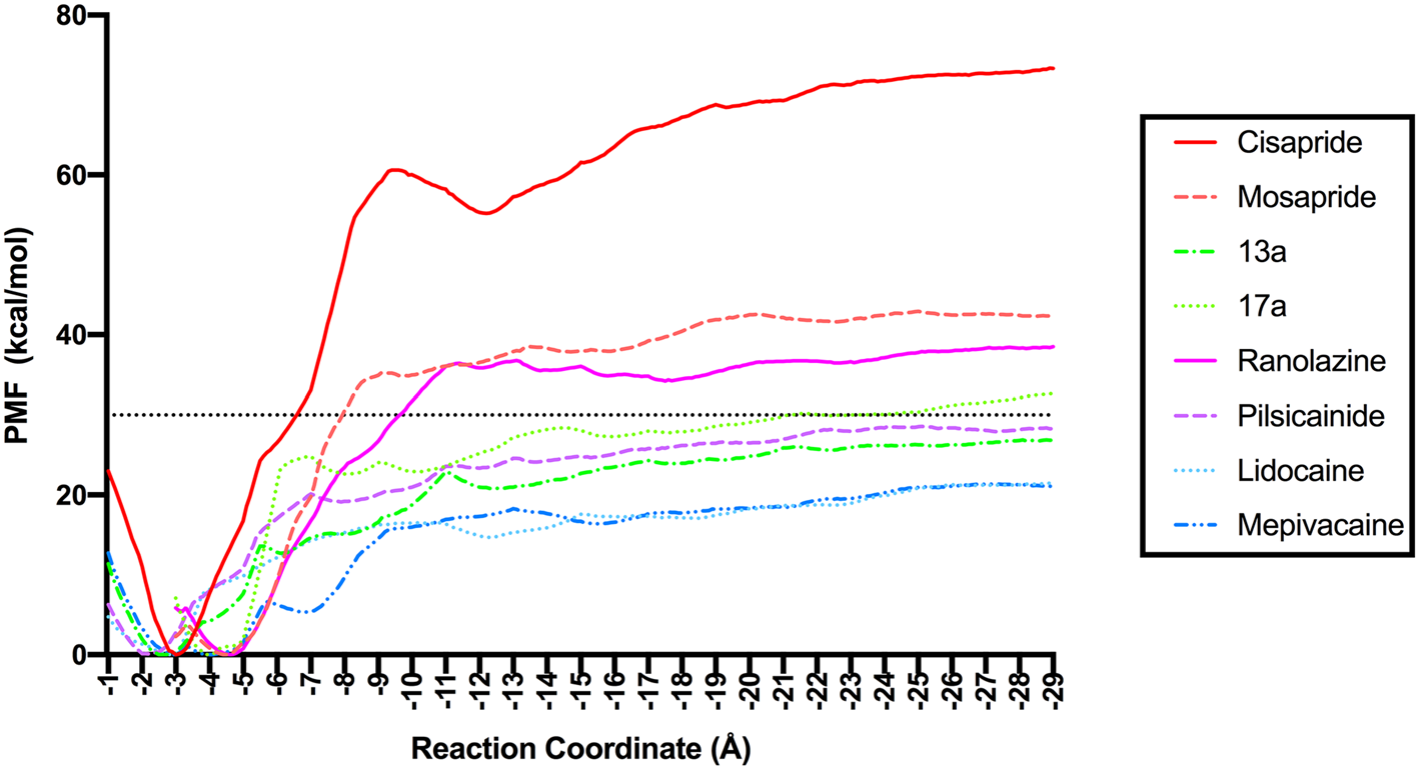
Potential of mean force for unbinding the drug (along the Z-direction with the chosen reaction coordinates) estimated from a 10 ns ABF simulation is shown. Also refer to supplementary Fig. 1 and 2 for the PMF profiles of drugs estimated from 1 ns simulation.

Initially, the PMF profiles were constructed for all five poses of every drug-hERG complex using a 1 ns ABF simulation for each window (supplementary Fig. 6 and 7). The PMF profiles of these poses were compared and the pose with the highest PMF profile (i.e., plateauing at larger energy ranges) was selected as the best pose. By adapting this strategy (supplementary Fig. 6 and 7), the best binding poses for each drug-channel complex was short-listed for further analysis. We found that the best binding poses identified from ABF simulations were in most cases consistent with those obtained from the MM-GBSA binding free energies (supplementary Table 2). Subsequently, the best poses (i.e., those with the highest PMF profiles) were studied again using ABF simulations, however, with a much larger simulation time per window (i.e. 10 ns per window). The relative ranking and low-energy states (favourable binding modes) of the drugs were analyzed using the PMF profiles constructed from these trajectories (Fig. 4).

The PMF profiles obtained from the 10 ns/window ABF simulations showed a clear differentiation of the small molecule analogs by their affinities and the predicted relative ranking of the drugs also agreed with the known experimental data^32–37^. Cisapride had the highest PMF with a ΔG_(PMF)_ corresponding to ~ − 72 kcal/mol (calculated from the depth to the plateau level at − 25 Å (see supplementary Fig. 8), followed by mosapride with a ΔG_(PMF)_ of ~ − 42 kcal/mol and ranolazine with a ΔG_(PMF)_ corresponding to ~ − 38 kcal/mol. The PMF profiles of 13a and 17a plateaued at ≤ − 30 kcal/mol, followed by pilsicainide with ~ − 28 kcal/mol. Both lidocaine and mepivacaine had ΔG_(PMF)_ ≥ − 20 kcal/mol, which agreed with their low affinities with the hERG channel. Analyzing the PMF profiles, it is clear that only three compounds, cisapride, mosapride and ranolazine exhibited free energies below − 30 kcal/mol, while all other compounds were above this threshold. This suggests that this two-stage ABF protocol is able to distinguish compounds with narrow ranges of inhibition and also effectively discern compounds above and below a 10 μM range. Despite the fact that ABF method requires longer simulation time to achieve convergence, we found that the 10 ns scale was sufficient to attain efficient classification of the compounds by their affinity. We performed multiple replicates of 10 ns per window ABF simulation (supplementary Fig. 9) and the overall trend were same among the replicates. Further, we performed the ABF simulations with 5–10 ns/per window and the PMF profiles from these simulations showed a converging trend with similar minima (supplementary Fig. 10). Since our intention was to use this workflow as a quick in silico screening protocol for predicting the hERG liability and to use the predicted binding mode as a blueprint for guiding medicinal chemistry optimization, we did not extend our simulation time scale. Nevertheless, if more computational resources are affordable, the use of longer simulation times is always recommended.

### Mode of binding of drugs to the hERG channel

From the calculated PMF profiles shown in Fig. 4 it is apparent that there is an initial decrease in the energy of the free energy landscape for all compounds, after which the free energy started to increase towards a plateau. This indicates that the initial poses suggested by docking simulations are not optimal, as their energies are not occupying the lowest energy positions. Nevertheless, the proximity of these starting docking locations to the identified minima suggests that the docking predictions were not far from the correct poses. A low energy position in the PMF landscape suggests that the ligand-channel complex relaxed and visited a low energy binding pose along the RC, which was more preferred by the compound compared to the original pose obtained from preliminary docking. The pose related to this energy minimum, therefore, can be considered as the favourable binding mode of drugs against hERG. For all drug-hERG complexes, the binding mode obtained from the ABF simulations showed less than 2 Å root mean square (RMS) deviation from the post-MD snapshot (i.e., the initial structure in ABF, see supplementary Fig. 11). This suggests that MD relaxation is important to account for the effects of binding-induced conformational changes for both the protein and the drug. The molecular interactions stabilizing the binding modes for all compounds were identified from their corresponding PMFs and are described below in detail.

**Figure 5:**
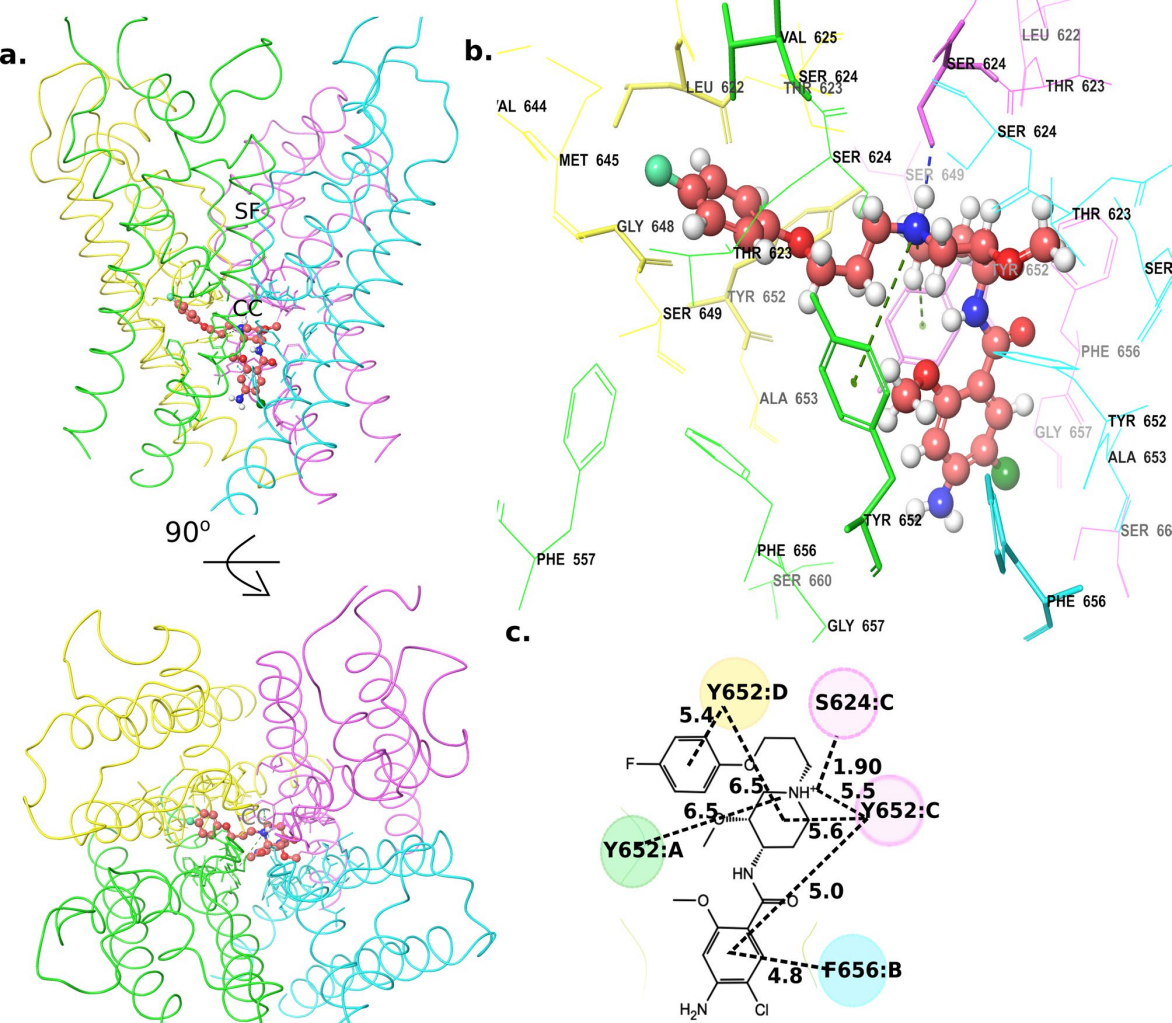
Cisapride low-energy binding mode (**a**) Structure of the Cisapride-bound channel side view with the extracellular side on the top and 90° rotated extracellular view. (**b**) A zoomed-in view of the cisapride bound to the central cavity. (**c**) A 2D representation of cisapride interactions with the residues in the central cavity. (Numbers show the distance between the two groups in angstrom). *SF* selectivity filter, *CC* central cavity.

Cisapride presented an inverted “L”-shaped binding mode against hERG, in which the 4-fluorophenol moiety and the propyl linker were placed perpendicular to the channel axis and the rest of the molecule lied parallel to the channel axis (Fig. 5a–d). The 4-fluorophenol group (the head group) occupied a lateral hydrophobic pocket between the two neighbouring subunits (chains D and A) below the selectivity filter, forming hydrophobic interactions with residues (TYR652, VAL625, GLY648, and LEU622) from both chains. The piperidine moiety (the middle ring) was placed at a position such that its cationic nitrogen group is well-positioned just beneath the potassium-binding site in the selectivity filter and formed a strong hydrogen bond (H-bond) with SER624 (chain C) and cation-π interactions with two tyrosine residues (TYR652) from chains A and C. As expected, the strong electrostatic potential observed in the cryo-em structure seem to play an important role in positioning the drug molecule. The terminal amide-ring (the tail group) of cisapride established vdW interactions with the side chain ring of PHE656 from chain B. These interactions between cisapride and TYR652 residues (from chains A, C, and D) and PHE656 (from chain B) remained stable during our MD simulations (supplementary Fig. 12), suggesting their importance in forming a high affinity complex. It is worthy a note that the head group in the initial docked pose of this complex neither occupied the hydrophobic pocket nor showed any significant contact with the PHE656 residue (supplementary Fig. 11). However, MD and ABF simulations allowed the system to relax and provided the lateral hydrophobic pocket access to the head group and enabled reorientation of PHE656 residue to form stable interactions with the tail group. Further, we also noted that the pose without access to the hydrophobic pocket (pose 4 in supplementary Fig. 1, 6) showed lower PMF, even though it shared the same energy well as that of the best pose (supplementary Fig. 13). This confirms the significance of the hydrophobic pockets’ reported by Wang et al.^25^, in drug binding and hERG blockade.

The low-energy binding mode from the ABF can be referred to as a ‘three-point’ anchoring mode, where the two terminal ring substructures of cisapride were anchored to the two diagonal chains D (inside the hydrophobic pocket) and B (near the intracellular gate); whereas, the middle ring was anchored to chain C (via H-bonds and cation-π interactions). By adapting this three-point anchored binding mode within the hERG central cavity, cisapride was able to completely obstruct the channel pore, which explained its high binding free energy from our PMF calculations and its nanomolar range hERG activity. This also agrees with a previous study^47^, based on tandem dimer experiments, that confirmed the roles of two adjacent TYR652 residues and a PHE656 residue in drug binding to hERG.

**Figure 6:**
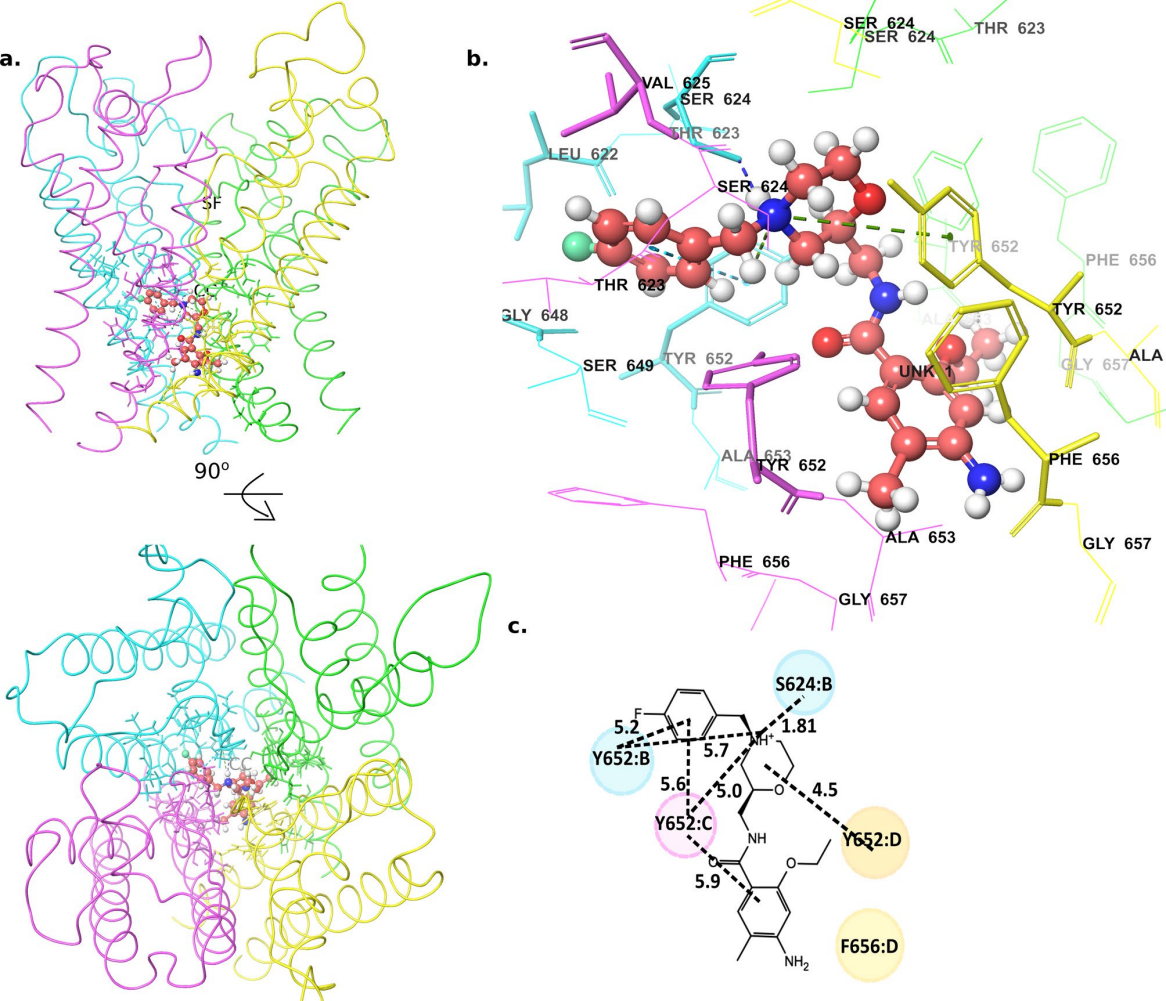
Mosapride low-energy binding mode (**a**) Structure of the mosapride-bound channel side view (top) and 90° rotated extracellular view (bottom). (**b**) A zoomed-in view of the mosapride bound to the central cavity. (**c**) A 2D representation of mosapride interactions with the residues in the central cavity. (Numbers show the distance between the two groups in angstrom). *SF* selectivity filter, *CC* central cavity.

Unlike cisapride binding mode that showed a larger deviation from its’ initial docked pose, the binding mode of mosapride and other analogs did not deviate much from their initial conformation (supplementary Fig. 14). On the other hand, similar to the parent compound, the analogs of cisapride in this study, mosapride, 13a and 17a, also presented inverted L-shaped binding modes (Fig. 6a–d, supplementary Fig. 15). While the low-energy modes seem relatively similar in their orientation, there were some significant differences observed in their channel interactions related to the anchor points that can explain their weaker affinities with hERG. In the binding mode identified for mosapride, its fluorophenyl ring (the head group) occupied the hydrophobic pocket between the adjacent chains B and C. However, due to a much shorter methylene linker in mosapride (against a longer propyl linker in cisapride), the fluorophenyl ring was not able to bury deep into the hydrophobic pocket, as was seen in cisapride (Fig. 6a–d). The protonated nitrogen group in the morphiline moiety of mosapride established H-bonds with SER624 (chain B) and cation-interactions with TYR652. Nevertheless, the tail ring (i.e., amide ring) in mosapride was separated from the PHE656 residues in all chains by at least a 7 Å distance, symbolizing no significant interactions with these residues (see supplementary Fig. 12). This agrees with the inhibition we observed in the mutant systems, for example, at 1 μM concentration of the drugs, cisapride showed ~ 80% reduction in the inhibition of F656A mutant when compared to the WT; while mosapride showed only 2% reduction in the inhibition of F656A mutant when compared to the WT (Table 2). Thus, in the predicted binding mode for mosapride, the head group (i.e., flurophenyl ring) was weakly anchored to the chains B/C and the tail group was not able to form interactions with any of the diagonal chains. This explained the weak affinity of mosapride to the hERG channel.

**Table 2:** Percent current inhibition of WT and mutant hERG channels by various compounds.

		hERG _WT_	hERG _Y652A_	hERG _F656A_
Compound	Concentration (µM)			
**Cisapride**	1	98.60% ± 1.40	7.70% ± 0.69	18.78% ± 5.69
	3	99.41% ± 0.59	39.10% ± 2.27	38.66% ± 8.08
	5	99.89% ± 0.11	57.70% ± 3.91	59.61% ± 7.46
	10	100.00% ± 0.00	91.72% ± 5.64	75.51% ± 13.26
**Mosapride**	1	13.07% ± 3.47	7.13% ± 3.47	10.99% ± 1.77
	3	33.48% ± 2.66	13.58% ± 2.28	12.45% ± 0.29
	5	44.96% ± 1.27	20.57% ± 3.01	16.03% ± 8.18
	10	68.74% ± 1.83	31.30 ± 3.65	25.71% ± 5.72
	30	92.45% ± 7.55	66.85% ± 12.66	34.35% ± 3.53
	100			40.21% ± 5.60
**Ranolazine**	10	42.27% ± 2.12	2.40% ± 0.76	4.39% ± 0.24
	30	76.97% ± 2.85	10.13% ± 0.48	10.86% ± 5.72
	100	97.36% ± 0.89	24.62% ± 2.64	21.37% ± 7.80
	300	100.00% ± 0.00	64.00% ± 6.08	37.74% ± 11.09
	500	100.00% ± 0.00	84.56% ± 4.88	49.62% ± 8.87
**Lidocaine**	10	2.99% ± 1.19	3.03% ± 1.77	5.49% ± 2.75
	30	7.79% ± 1.00	5.53% ± 2.52	8.10% ± 3.59
	100	19.13% ± 1.75	11.94% ± 0.64	15.08% ± 1.78
	300	42.21% ± 4.03	25.66% ± 0.54	26.63% ± 3.49
	500	56.06% ± 3.55	45.01% ± 2.90	35.48% ± 5.19
**n = **		3	3	2 to 6

In the case of compounds 13a and 17a, both compounds have a head group made of a five membered ring (pyrrole in 13a and pyrroline in 17a) replacing the fluorophenyl ring seen in cisapride. As a result, both compounds were unable to fill the hydrophobic pocket between the neighbouring chains under the selectivity filter. Similar to mosapride, the tail groups in 13a and 17a also did not interact with PHE656 (supplementary Fig. 15). Thus, their binding modes suggest that they were anchored only via their middle ring (i.e., through H-bond and/or cation-π interactions), which in turn resulted in a weak affinity towards the hERG channel.

**Figure 7:**
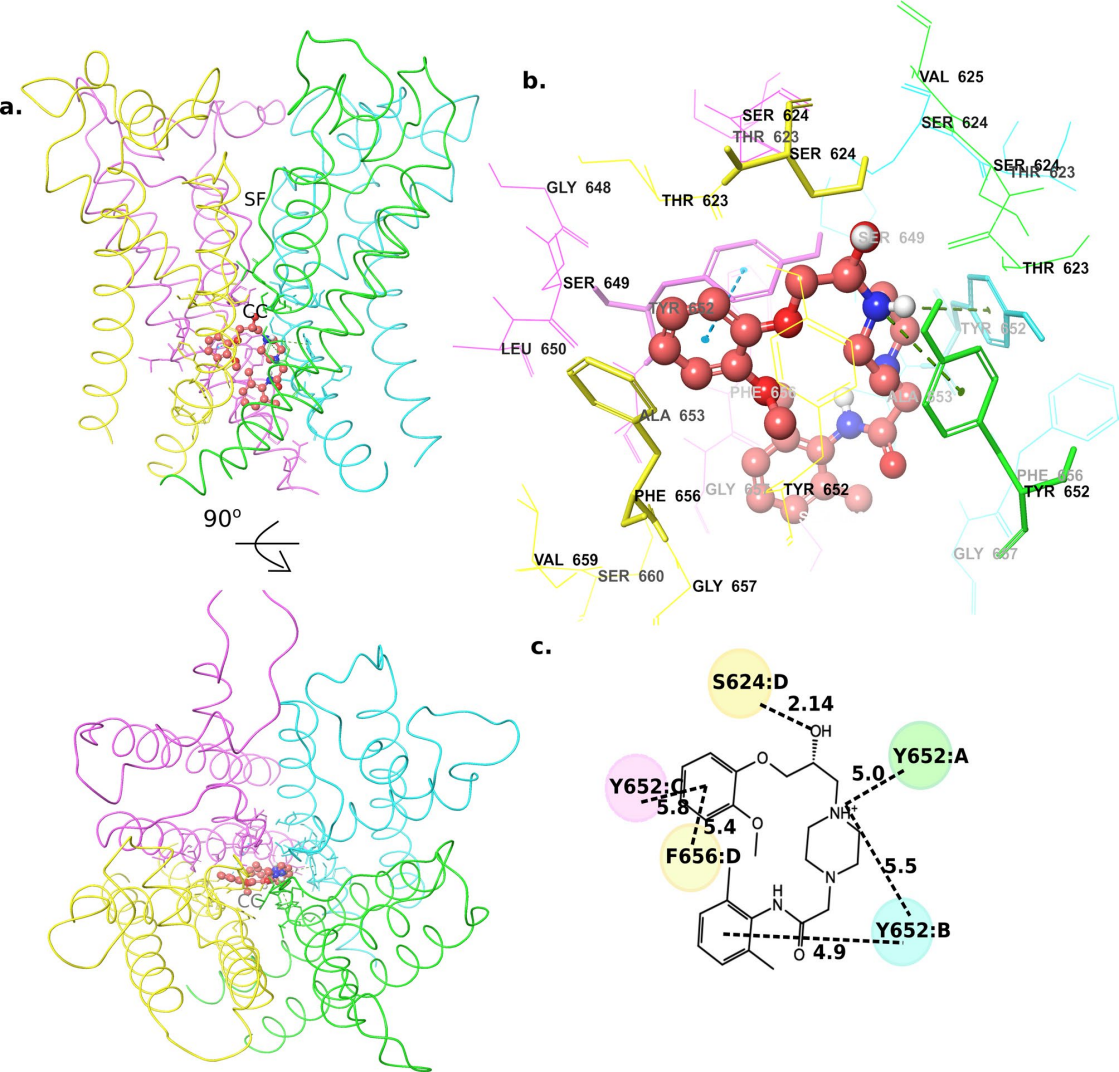
Ranolazine low-energy binding mode (**a**) Structure of the ranolazine-bound channel side view (top) and 90° rotated extracellular view (bottom). (**b**) A zoomed-in view of the ranolazine-bound to the central cavity. (**c**) A 2D representation of ranolazine interactions with the residues in the central cavity. (Numbers show the distance between the two groups in angstrom). *SF* selectivity filter, *CC* central cavity.

**Figure 8:**
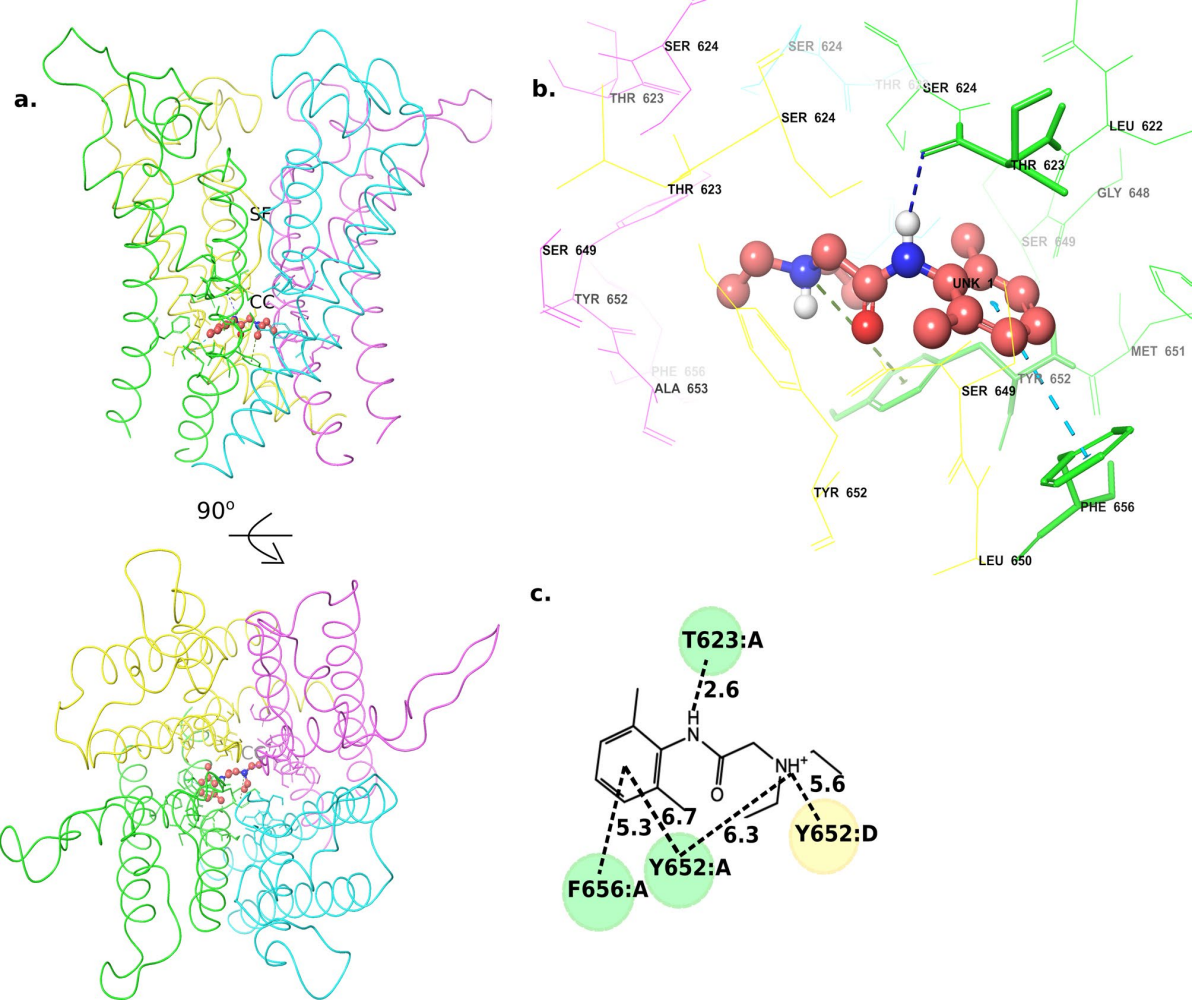
Lidocaine low-energy binding mode (**a**) Structure of the lidocaine-bound channel side view (top) and 90° rotated extracellular view (bottom). (**b**) A zoomed-in view of lidocaine-bound to the central cavity. (**c**) A 2D representation of lidocaine interactions with the residues in the central cavity. (Numbers show the distance between the two groups in angstrom). *SF* selectivity filter, *CC* central cavity.

The structure of ranolazine includes three rings, a methoxyphenol moiety (the head group), a piperazine moiety (the middle ring) and a dimethylbenzene ring (the tail group). The low-energy binding mode identified for ranolazine showed notable difference from its initial docked pose. The head group moved upwards towards the hydrophobic pocket during the MD equilibration and tail group straightened up breaking the intramolecular stacking found in the initial pose (supplementary Fig. 16). In the predicted binding mode of ranolazine, the methoxyphenol group is placed near the hydrophobic cavity, similar to cisapride. However, due to the presence of a methoxy moiety, the head group is not able to fit into the lateral hydrophobic cavity within chains C and D (Fig. 7a–d); instead, it is placed outside the cavity and interacts with PHE656 (chain D) and TYR652 (chain C) (supplementary Fig. 17). The hydroxyl group present in the linker is within H-bonding distance to SER624 and the cationic nitrogen group within the piperazine ring is anchored through cation-π interactions with two tyrosine residues, namely the two copies of TYR652 from chains A and B. Nevertheless, the ranolazine tail group does not make stable interactions with PHE656 residue from either of the diagonal chains, as was shown in the cisapride case. Instead, it formed a π**–**π interaction with TYR652 from chain B. The head group in ranolazine, although had restricted access to the lateral hydrophobic cavity, showed stable interactions with the PHE656 residue. The notable drop in the inhibition in F656A mutant channel (Table 2 and ref.35) is probably due to this stable interaction of the head group with PHE656 residue. Similarly, T623A and S624A mutation was previously reported to cause ~ 19- and ~ 8-fold reduction in inhibition, respectively, when compared to the WT channel^35^. Our MD simulations showed H-bond interactions between ranolazine and the two residues THR623 and SER624 residue (supplementary Fig. 17). The weaker interactions (~ 5–7 Å) of the tail group with PHE656 residue together with the weak anchoring of the head group at the hydrophobic pocket probably results in the weaker affinity of ranolazine. In the case of other low-affinity molecules (i.e. lidocaine, mepivacaine and pilsicainide), they have only one or two ring substructures, which included a cationic nitrogen containing group and a dimethylbenzene (tail group). Lidocaine moved towards the hydrophobic pockets during the MD simulations with its’ aromatic ring facing towards the central cavity (Fig. 8a–d, supplementary Fig. 18). The predicted binding modes for these compounds show that their cationic nitrogen group is anchored through cation–π interactions with TYR652 and their tail group is either placed parallel (in mepivacaine supplementary Fig. 19) or perpendicular (in lidocaine and pilsicainide) to the channel axis (Fig. 8a–d, supplementary Fig. 19). Given their small structures and their inability to engage with all the three anchor sites in hERG, it is not surprising that these compounds exhibit weak/no affinity to the hERG channel.

### Effects of anchor site mutations on drug binding and hERG current

The predicted modes of binding for the tested compounds highlight three sites in the hERG channel that play important roles in drug binding. Particularly, the three residues, PHE656, TYR652 and SER624, were found to be involved in anchoring drugs to these sites through cation-π, π**–**π and H-bond interactions. Of these residues, we tested the effects of mutating TYR652 and PHE656 to alanine (i.e., Y652A and F656A) on drug binding and K^+^ ionic current from hERG, using fluorescent polarization-based (FP) binding assay and patch clamp experiments.

**Figure 9:**
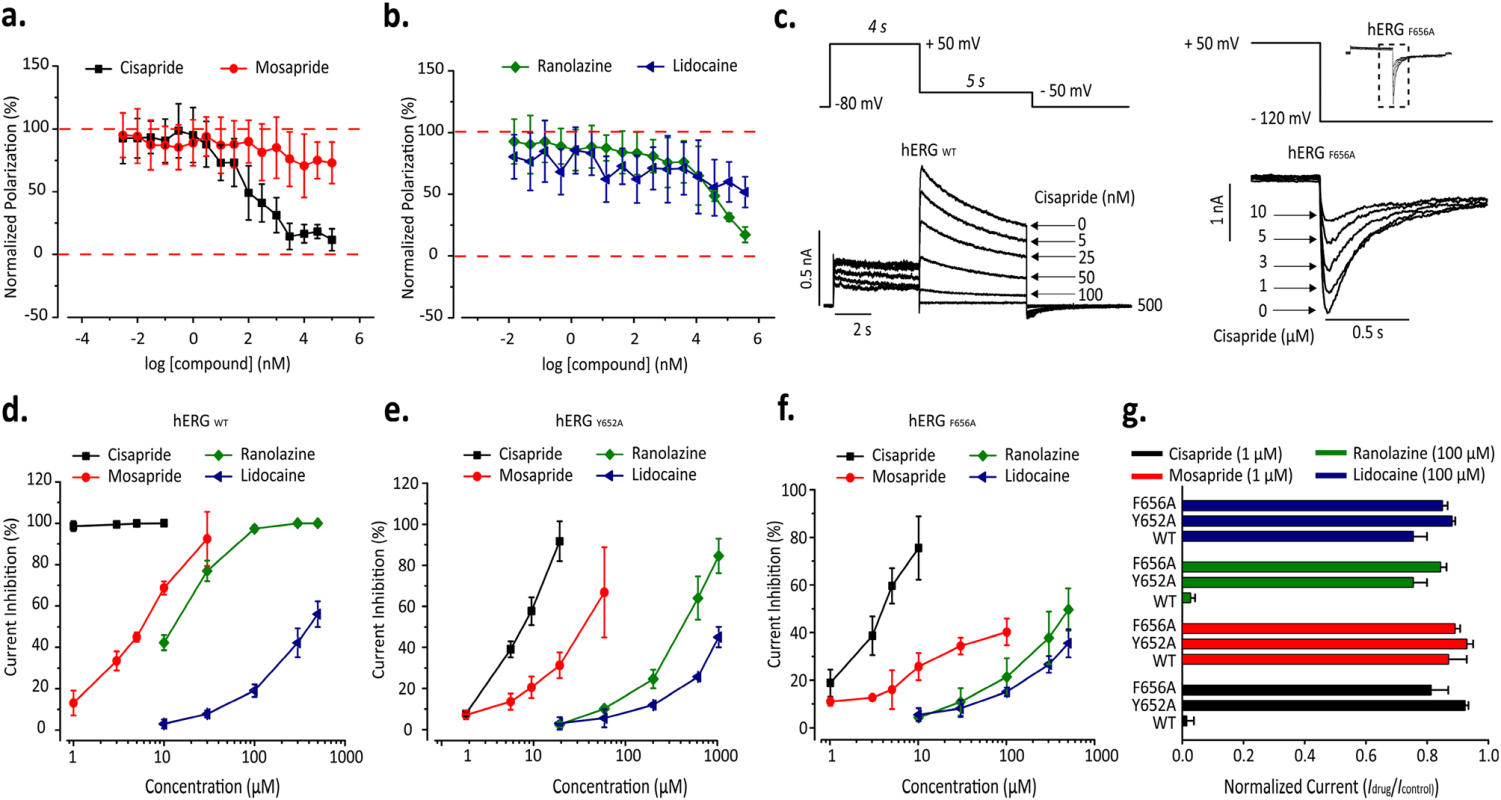
Binding and electrophysiology analysis of the hERG channel. Competitive binding of (**a**) Cisapride and Mosapride, and (**b**) Ranolazine and Lidocaine with the tracer (that shares the same binding site as E−4031), measured using a hERG polarization assay kit (n = 4). (**c**) Left, Representative current traces of a concentration-dependent drug block (cisapride, nM) of the wild type (hERG_WT_) channel recorded in a 5 mM K^+^ external solution using manual patch clamp methods. Right, Representative current traces of a concentration-dependent drug block (cisapride, µM) of the F656A mutant (hERG_F656A_) channel recorded in a 135 mM K^+^ external solution using manual patch clamp methods. (**d**–**f**) Concentration-dependent effects of drugs (Cisapride, Mosapride, Ranolazine and Lidocaine) in hERG_WT_ (automated patch data)_,_ hERG_Y652A_ (automated patch data) and hERG_F656A_ (manual patch data), respectively. (**g**) Normalized current (I_drug_/I_control_) measured after steady-state block by the drugs at the specified concentrations for WT and the two mutants Y652A and F656A. All data are reported as mean ± SD.

We initially tested the binding of four drugs, namely cisapride, mosapride, ranolazine and lidocaine, to the wildtype (WT) hERG channel using an FP assay. The results show that cisapride and ranolazine exhibited dose dependent activities, where the former had much higher affinity to hERG than the latter. The IC50 values of cisapride and ranolazine were estimated to be 184.9 nM and 126.69 μM, respectively. On the other hand, mosapride (a cisapride-analog) and lidocaine (a ranolazine-analog) exhibited much weaker affinity to WT hERG (Fig. 9a,b). The overall binding trend from our FP assay is in good agreement with the binding affinity data from the literature^17^ as well as with our predicted free energies from the ABF simulations.

Next, we measured hERG current upon binding of drugs to hERG_WT_, hERG_Y652A_ and hERG_F656A_ in HEK stable cell lines using whole-cell patch clamp experiments. The hERG_WT_ channel was recorded in a standard 5 mM K^+^ external solution for manual patch clamp experiments (Fig. 9c) or an extracellular ringer’s solution for automated patch clamp experiments. Due to inadequate current through hERG_F656A_ in low mM K^+^ external solutions, as previously reported^27^, drug-induced block of the hERG_F656A_ mutant was recorded in a 135 mM K^+^ external solution using manual patch clamp methods (Fig. 9c). Cisapride inhibited WT hERG current with an IC_50_ of 33.42 nM and completely inhibited the channel at concentrations of 1 μM and higher, which reflects its strong affinity to hERG, correlating with our binding free energies (from PMF) and our FP-binding assay (Figs. 4 and 9a,b). In the case of mosapride and ranolazine (known weak binders), both compounds exhibited a dose-dependent current inhibition in WT hERG, where 50% of current inhibition (IC_50_) was achieved with 5.36 μM for mosapride and with 12.54 μM for ranolazine. Lidocaine exhibited the weakest effect on WT hERG with an IC_50_ > 500 μM in our patch clamp assay (Fig. 9d–f).

Testing the compounds in the Y652A and F656A mutants did show some dose-dependent inhibition of hERG current, however, unlike in the WT system, none of the drugs, at the same range of concentrations tested with WT, were able to completely inhibit the hERG current of these mutants. The IC_50_ values of all drugs dropped significantly in the mutant systems. For example, the IC_50_ value of cisapride in the Y652A system was estimated at 3.89 μM, which is much weaker compared to its activity in WT hERG (*i.e.,* 100% inhibition at 1 μM). Similarly, the IC_50_ values of mosapride and ranolazine in the Y652A mutant were estimated at 17.4 μM and 195 μM, respectively, which have several fold reduction in activity compared to WT hERG (IC_50_ values of 5.36 μM of mosapride and 12.54 μM of ranolazine).

In order to understand the implications of mutations on the tested drugs, we compared the amount of current seen in the WT to those from the mutants (Y652A and F656A) at a selected drug concentration within the analogues. The normalized current (I_drug_/I_control_) was obtained for both cisapride and mosapride at 1 μM concentration. At this concentration, cisapride completely inhibited the WT hERG current, while inhibiting only about 20% or less of the currents from the mutants (Fig. 9g). This validates our binding mode prediction for cisapride against hERG, in which we suggested that cisapride interacted strongly with both Y652A (via its head group) and F656A (via its tail group). Thus, mutating these residues resulted in weak/loss of binding of cisapride to hERG, which led to poor inhibition of current in the mutant hERG systems. On the other hand, a 1 μM concentration of mosapride inhibited a very small percentage of the hERG current in both WT and mutants, which was expected as this compound already exhibits much weaker affinity to WT hERG (Fig. 9g). Similarly, the normalized current for both ranolazine and lidocaine from set 2 was measured at 100 μM concentrations. At this concentration, ranolazine inhibited ~ 90% of WT hERG current, but only 25% of Y652A current and 10% of F656A current (Fig. 9g). The slightly better inhibition by ranolazine seen in the Y652A mutant compared to that of F656A showed a strong agreement with the binding mode predicted for ranolazine in this study. We predicted that the head group (i.e., methoxyphenol moiety) of ranolazine interacted with TYR652 and PHE656 residue, whereas the tail group (dimethylbenzene) did not make stable interactions with PHE656, as shown in the cisapride binding mode. Finally, 100 μM of lidocaine inhibited ~ 25% of WT hERG current, which reflected its poor affinity towards hERG. In the case of the mutant channels, 100 μM of lidocaine had very little impact (about 10%) on the ionic current (Fig. 9g). Therefore, in summary, our experiments confirm the importance of the two residues, TYR652 and PHE656, on the binding modes and activities of the drugs studied in this work.

### Effects of drug binding on the π–π networks in hERG

Our analyses from this work suggest that aromatic residues at the hERG central cavity such as TYR652 and PHE656 are involved in drug binding. It is known that there are a number of aromatic residues in hERG that form π**–**π stacking interactions to stabilize the conformation of the channel pore. Some of these residues have been identified to play important roles in modulating channel blockade by drugs either through direct interactions with these drugs or through allosteric mechanisms. For example, a previous study^21^ identified that PHE619 in the pore helix of hERG exhibits moderate effects on drug-induced channel blockade, whereas, another aromatic residue, namely PHE557, from the S5 helix is suggested to allosterically modulate the channel inhibition by drugs^21^. Thus, it might be interesting to study the effects of drug binding on different π**–**π stacking interactions within the hERG cavity. For this purpose, we analyzed all π**–**π interactions in the apo-state of hERG using the residue interaction network generator (RING2.0) program^48^, and studied how these interactions were affected upon binding to drugs, such as cisapride, mosapride, ranolazine and lidocaine.

**Figure 10:**
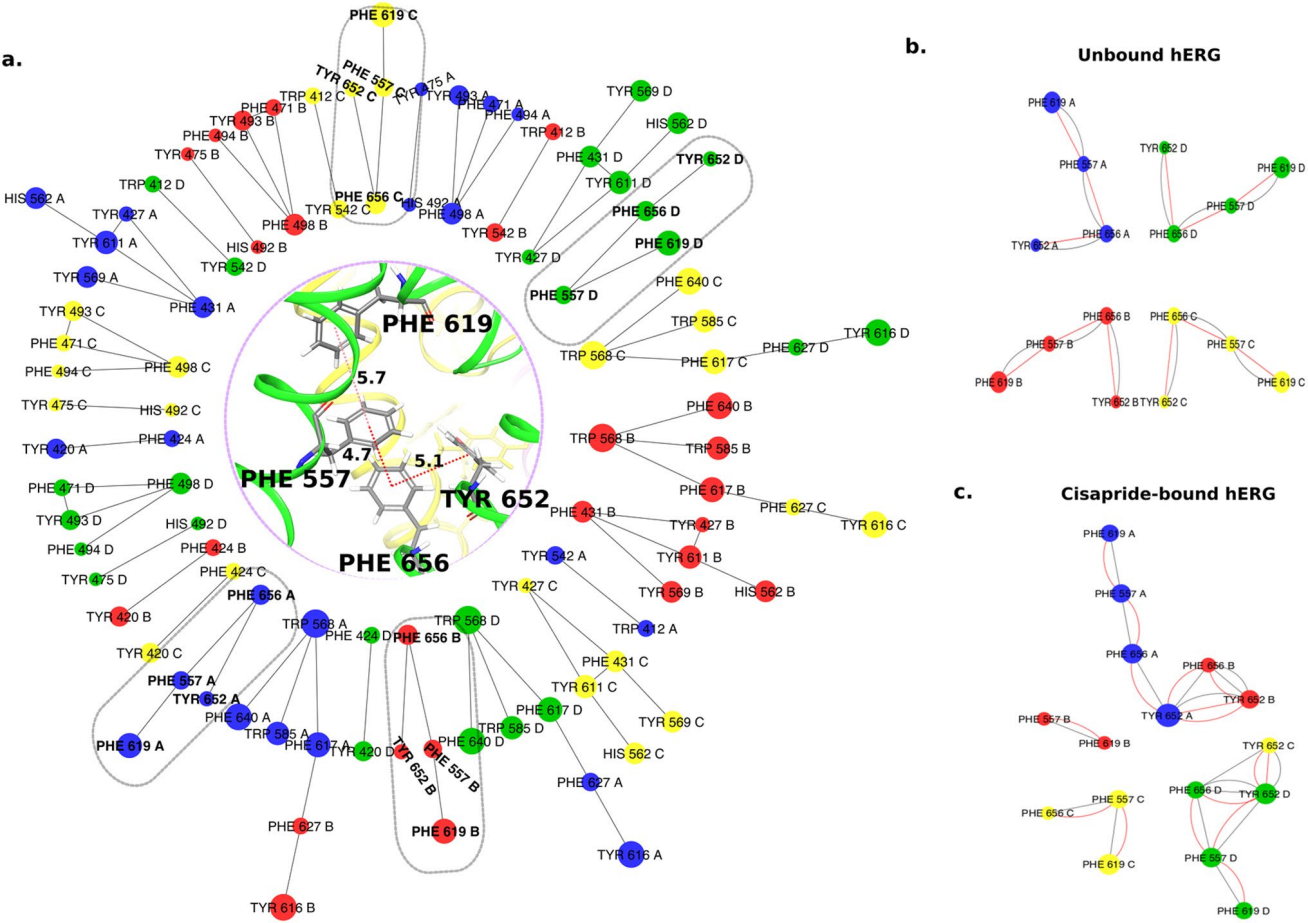
Network of Pi–Pi interactions (**a**) Pi-networks found in the Cryo-EM structure of the hERG channel (PDB: 5VA1). The pi-network involved in the key drug binding residues (TYR 652, PHE 656, and PHE557) from all chains are marked in dotted ovals; The orientation of the residues (marked in the oval) in the cryo-em structure of the hERG channel is given at the center. (**b**) a zoomed-in view of the Pi-network in an unbound channel (among the residues marked in the oval) (**c**) Rearrangement of the Pi-network when Cisapride is bound as obtained from the lowest energy binding mode structure. Red solid lines indicate pi-pi contact.

Figure 10a shows a network of π**–**π interactions present in the cryo-EM structure of the apo hERG channel. As shown in this figure, the channel is rich in π**–**π stacking interactions. We mainly focused on a chain of stacked aromatic residues formed by TYR652-PHE656-PHE557-PHE619 in all four chains (Fig. 10b), as this network was linked to the ligand anchoring sites that we characterized from our binding-mode analyses. Similar analyses on the low energy structure of the drug-bound complexes) revealed that the π-network was affected when drugs were bound to the channel (Fig. 10, supplementary Fig. 20). For example, as discussed earlier, cisapride was found to interact with TYR652 from chains A/D/C and PHE656 from chain B. Binding of cisapride with PHE656 in chain B, broke its π**–**π interaction with PHE557 in this chain (Fig. 10c, supplementary Fig. 21). Similarly, binding of cisapride with TYR652 from chain C, cleaved this residue from its π–π association with PHE656 in this chain (supplementary Fig. 21). Apart from breaking these native π–π networks, cisapride also resulted in the formation of new π–π connections, such as linking the TYR652-PHE656 network from chain B with the aromatic network in chain A. Similar rearrangement of π**–**π networks of TYR652-PHE656-PHE557-PHE619 was also induced by ranolazine (supplementary Fig. 20). In the proposed binding mode for ranolazine, it was found to interact with TYR652 from chains A/B/D. As a result, the binding of ranolazine resulted in the union of the π**–**π networks from chains A/B/D, which were otherwise independent of each other in the unbound protein (Fig. 10a). The binding mode analyses of Mosapride showed that it interacted with TYR652 from chains B/C/D. Therefore, as expected, the π-network of TYR652-PHE656-PHE557-PHE619 from chains C and D were fused together. Finally, lidocaine, a weak hERG blocker, was found to bind to TYR652 from chain A. As a result, the π**–**π interaction of TYR652 with PHE656 in chain A was disrupted (supplementary Fig. 21*)*, and the former was connected to the π-network of chain B (supplementary Fig. 20).

It is well known that the some of the drugs used in this study show higher affinity to the inactivated state of the channel. Since the π-network correspond to residues from both the central cavity (PHE656, PHE557, and TYR652) and near the selectivity filter (PHE619), the rearrangement could probably be a feature of the inactivated state of the channel. Mutagenesis experiments and more simulations are required to gain deeper understanding on this aspect of drug-induced effects on native π-networks in hERG.

## Conclusion

Over the years, substantial effort has been made to understand the structural aspects of drug-induced hERG blockade. Both computational and experimental analyses have confirmed that many of the LQTS-causing drugs bind to the central cavity present in the pore of the hERG channel. Two key residues, TYR652 and PHE656, were identified to play a key role in the drug-induced blockade of the hERG channel. Nevertheless, the growing numbers of drugs and the diversity in their chemotypes pose a serious challenge in understanding the structural features of the channel blockade.

Here, we employed an in silico structure-based workflow to predict hERG liability of drugs and to understand the molecular interactions of the drug-channel complexes. We tested our workflow using small molecule analogs from two classes of drugs, cisapride and ranolazine. Our results show that the docking-based scoring was not accurate enough to classify the analogs by their affinities. MD-based MM-GBSA rescoring showed a significant improvement in predicting the compound’s hERG liability yet lacked precision in compounds with narrow affinity-variations. By using ABF simulations, we were able to achieve an improved precision in differentiating structurally similar analogs and ranking them by their affinities. Our analyses of the PMFs were useful to identify the low energy-binding modes of these drugs in the hERG channel. The binding mode of the drugs suggested that a small molecule binding to three anchor points would have greater affinity to the channel. We confirmed the role of the key interacting residues by measuring the effects of drugs towards the channel current in both WT-hERG and mutated hERG channels. Our π**-**network analyses on the complex structures showed that drug binding results in the disruption and/or rearrangement of the π**-**π network found between the aromatic residues (Tyr652, Phe656, Phe557) of the central cavity of the channel. Given the results, our workflow would not only be useful for predicting drug-mediated hERG liability but can also be used as a complementary protocol for designing functional groups when optimizing the drug-like leads to reduce their hERG liability.

## Methods

### hERG channel structure preparation

The cryo-EM structure of the hERG channel was obtained from the PDB database [PDB ID: 5VA1]^25^. Since the coordinates of some of the extracellular loops were missing in the structure, the missing loops were built and refined using the Prime program of the Schrodinger suite^49^. The structure corresponding to the residues 398–668, which encompasses the transmembane domain region of the channel was used. The protonation states of the residues were assigned using the PROPKA program using a pH of 7^50^. The final structure was prepared and energy-minimized using the Impref utility in the protein preparation wizard in Maestro (Schrodinger LLC)^51^.

### Ligand preparation and docking protocol

The selected ligands were prepared using the Ligprep module of the Schrodinger suite^50^, and the ligand conformations were sampled using the Macromodel conformational search protocol. All sampled conformations were used as the input for the rosetta ligand docking protocol^52^. More than 500,000 docked complexes were obtained for each ligand from seven individual docking runs that covered a sampling radius of 15 Å from the centre of central cavity residues (Ser624, Tyr652 and Phe656). A 5 Å initial perturbation was applied to allow randomization of the ligand position. Top 100 poses from each of the seven runs, were obtained based on the total scores obtained for the protein–ligand complexes. The poses were clustered by applying a 2 Å threshold using a greedy algorithm-based clustering approach implemented in the ‘select_best_unique_ligand_poses’ module of the Rosetta package, and five best scoring pose were written for each docking run. The 35 docked complexes (5 poses * 7 independent runs) were sorted based on Rosetta’s interface delta_X energy^52,53^, which is the binding energy calculated as the difference between overall rosetta energy of the complex and the energies of the individual components. Redundant and symmetrical poses (2 Å threshold) were filtered out manually, and the top five poses with the best interface delta_X energy were taken for subsequent molecular dynamics (MD) simulations.

### MD System preparation and equilibration protocol

Five different complexes of drug bound hERG channel were embedded into a 120 X 120 Å POPC lipid bilayer using the Membrane plugin of the VMD software^54^. The solvate plugin of VMD package was used to solvate the membrane-embedded complexes of the drug-hERG channel with TIP3P water. The autoionize plugin was used to electro-neutralize the system and increase its’ ionic concentration to 150 mM of KCl. Each complex system included more than 140,000 atoms. The system was parameterized using the CHARMM36 forcefield^55^. The structural parameters for all the drugs were obtained from the Swissparam server^56^. Two-stage energy minimization of the membrane-embedded complexes of the drug-hERG channel was carried out. During the first stage, the protein, ligand and the lipid heads were fixed allowing the other components to relax for 250,000 steps. This stage was essential to remove any atom overlaps from improper packing of the membrane around the protein. In the next stage, a 1 kcal/mol.Å harmonic restraint was placed on the protein and ligand atoms, and the system was energy minimized for another 250,000 steps. Following the minimization, an all-atom equilibration was performed for 10 ns time scale with a 2 fs time step. The systems were equilibrated at 310 K temperature and 1 atm pressure, controlled by the Langevin thermostat and piston. A 12 Å cut-off with a switching distance and pairlist distance of 10 Å and 13.5 Å, respectively, was used for treating the long-range electrostatics using the Particle Mesh Ewald (PME) method. All the simulations were performed using the NAMD (v2.12) molecular dynamics software^57^.

### Molecular mechanics generalized born solvation area (MM-GBSA)

Binding free energies of the five different poses of the drug bound to the hERG channel were calculated using the MM-GBSA method implemented in the MMPBSA.py script^58^ of the AMBERTools^59^. The GBradii developed by Onufriev et al.^60^, was used for estimating the free energies. The binding free energy for each selected frame was computed using the following equation,

$$\Delta G= {\Delta E}_{MM}+ {\Delta G}_{solv}$$

The final binding free energies were averaged over twenty-one snapshots sampled from the last 8 ns of equilibration trajectories. ParmEd package^61^ of the AmberTools was used for converting the CHARMM topologies to Amber formats.

### Adaptive biasing force (ABF) simulation

The PMF of unbinding the bound conformation of the drug through the intracellular gate of the hERG channel (i.e., reaction coordinate) was estimated using the adaptive biasing force (ABF) simulations. In the ABF method, an adaptive biasing force is applied to counteract the energetic barriers along the reaction coordinate, which helps in the enhanced sampling of the free energy landscape. The reaction coordinate was defined as the distance between the center of mass of the protein and the center of mass of the bound drug along the Z-axis. The length of the reaction coordinate (~ 26–28 Å) is split into 13–14 multiple windows of 2 Å lengths, depending on the location of the bound drug. The upper and lower wall constants were set to 100 kcal/mol.Å^2^. The fullSample parameter was set to 1000 and the bin width was set to 0.1 Å to allow adequate sampling and smooth PMF. The coordinates of the windows were sampled from a steered molecular dynamics simulation (see supplementary text for details) of the equilibrated complex. Initially, all the windows were simulated for 1 ns time scale using the ABF method. The PMFs obtained for the five different poses of the drug were compared to find the pose with higher PMF. This pose was selected for an extended simulation where each window was simulated for 10 ns time scale. These simulations result in ~ 250 ns simulation for a drug, including the five selected binding poses in the hERG channel. Thus, the cumulative time scale of simulations performed in this study would account to ~ 2 μs.

### hERG polarization kit assay

Drugs were dissolved in 100% DMSO and their ability to compete with E-4031 and block the hERG channel were evaluated under 1.4% final DMSO concentration using the Predictor™ hERG Fluorescence Polarization Assay kit (Life Technologies, Burlington, ON, Canada). All measurements were made according to the manufacturer’s instruction. Fluorescence polarization was measured using PerkinElmer EnVision^®^ Multilabel reader (PerkinElmer, Woodbridge, ON, Canada). The IC_50_ values of the drugs were calculated using GraphPad Prism 7.03 (GraphPad Software, San Diego, CA) from their predicted dose–response four parameter logistic curve with normalization, and with top and bottom constraints applied as suggested by the kit’s user manual.

### Manual-patch clamp electrophysiology

The whole cell-patch clamp method was used to record current from HEK cells stably expressing hERG_WT_, hERG_Y652A_ and hERG_F656A_. hERG-HEK cells were cultured and maintained in biolite 25 cm^2^ flasks (Thermo Fisher) in a 5% CO_2_ incubator at 37 °C in DMEM-high glucose (D5671; Sigma) supplemented with 10% FBS, 1% penicillin/streptomycin and (0.4 mg/ml) G418 for maintenance. 12 h prior to recording, cells were split onto sterile glass coverslips in 6-well plates. Patch pipettes were manufactured from soda lime capillary glass (Fisher), using a Sutter P-97 (Sutter Instrument) puller. When filled with recording solutions, pipettes had a tip resistance of 1–3 MΩ. Recordings were filtered at 5 kHz, and sampled at 10 kHz, with manual capacitance compensation and series resistance compensation at 80%. Recordings were stored directly on a computer hard drive using Clampex 10 software (Molecular Devices). The standard 5 K^+^ external (bath) solution had the following composition (in mM): 135 NaCl, 5 KCl, 1 CaCl_2_, 1 MgCl_2_, 10 HEPES and was adjusted to pH 7.3 with NaOH. The high 135 K^+^ external (bath) solution had the following composition (in mM): 135 KCl, 2 CaCl_2,_ 1 MgCl_2_, 10 HEPES and was adjusted to pH 7.4 with KOH. The internal (pipette) solution had the following composition (in mM): 135 KCl, 5 EGTA, 10 HEPES and was adjusted to pH 7.2 using KOH. Current recordings in the standard 5 K^+^ bath solution were evoked with a depolarizing step to 50 mV for 4 s from a − 80 mV holding voltage. A repolarizing step to − 50 mV for 5 s was used to record outward hERG tail currents. Current recordings in the high 135 K^+^ bath solution were evoked with a depolarizing step to 50 mV for 4 s from a − 80 mV holding voltage. A repolarizing step to − 120 mV for 5 s was used to record inward hERG tail currents. Analysis of tail current amplitudes were used to measure current inhibition due to drug block. Patch clamp experiments were conducted at room temperature (22 ± 1 °C).

### Automated-patch clamp electrophysiology

Cells were cultured as described in the manual patch clamp electrophysiology method section. Recordings were obtained using the Ionflux 16 automated patch-clamp machine from FLUXION BIOSCIENCES. The external (bath) solution was an extracellular ringer’s solution: 1X Dulbecco’s Phosphate-Buffered Saline (DPBS) with calcium and magnesium (Corning, catalog # 21-030-CV) supplemented with 10 mM HEPES and adjusted to pH 7.4 (with NaOH) and 300 mOsm (with sucrose). The internal (pipette) solution had the following composition in (mM): 90 KF, 30 KCl, 2 MgCl_2_, 10 HEPES, 10 EGTA and was adjusted to pH 7.2 (with KOH) and 285 mOsm (with sucrose). Currents were evoked with a depolarizing step to 40 mV for 800 ms from a − 80 mV holding potential. A repolarizing step to − 40 mV for 1.2 s was used to record outward tail current.

## Supplementary information

Supplementary file1
