# Supplementary material for: A structure-based computational workflow to predict liability and binding modes of small molecules to hERG
Source: Sci Rep. 2020 Oct 1;10:16262. doi: 10.1038/s41598-020-72889-5 (PMC7530726; doi:10.1038/s41598-020-72889-5)
Supplement: Supplementary file 1 — Supplementary file1 [file 41598_2020_72889_MOESM1_ESM.docx]

**Supplementary Information**

**A Structure-Based Computational Workflow To Predict Liability And Binding Modes Of Small Molecules To hERG**

Subha Kalyaanamoorthy^1,5^ Shawn M. Lamothe^2^, Xiaoqing (Ervin) Hou^3,4^, Tae Chul Moon^1^, Harley T. Kurata^2^, Michael Houghton^3,4^, Khaled H. Barakat^1^

^1^ Faculty of Pharmacy and Pharmaceutical Sciences, University of Alberta, Edmonton, AB, Canada.

^2^ Department of Pharmacology, Faculty of Medicine & Dentistry, University of Alberta, Edmonton, AB, Canada.

^3^ Department of Medical Microbiology & Immunology, Faculty of Medicine & Dentistry, University of Alberta, Edmonton, AB, Canada.

^4^ Li Ka Shing Applied Virology Institute, University of Alberta, Edmonton, AB, Canada.

^5^ Department of Chemistry, University of Waterloo, Waterloo ON Canada.

**Supplementary Table 1:** RMSDs (in Å) of the five poses selected from docking

a. Cisapride

b. Mosapride

|  | **Pose 1** | **Pose 2** | **Pose 3** | **Pose 4** | **Pose 5** |
| --- | --- | --- | --- | --- | --- |
| **Pose 1** | **0.0** | 7.61 | 4.74 | 5.49 | 6.68 |
| **Pose 2** | 7.61 | **0.0** | 9.09 | 6.50 | 2.85 |
| **Pose 3** | 4.74 | 9.09 | **0.0** | 5.21 | 8.44 |
| **Pose 4** | 5.49 | 6.50 | 5.21 | **0.0** | 7.22 |
| **Pose 5** | 6.68 | 2.85 | 8.44 | 7.22 | **0.0** |

c. Ranolazine

d. Lidocaine

e. 13a

|  | **Pose 1** | **Pose 2** | **Pose 3** | **Pose 4** | **Pose 5** |
| --- | --- | --- | --- | --- | --- |
| **Pose 1** | **0.0** | 3.46 | 5.04 | 5.12 | 3.79 |
| **Pose 2** | 3.46 | **0.0** | 4.71 | 4.95 | 3.51 |
| **Pose 3** | 5.04 | 4.71 | **0.0** | 4.85 | 3.13 |
| **Pose 4** | 5.12 | 4.95 | 4.85 | **0.0** | 4.63 |
| **Pose 5** | 3.79 | 3.51 | 3.13 | 4.63 | **0.0** |

|  | **Pose 1** | **Pose 2** | **Pose 3** | **Pose 4** | **Pose 5** |
| --- | --- | --- | --- | --- | --- |
| **Pose 1** | **0.0** | 3.72 | 3.31 | 3.91 | 5.05 |
| **Pose 2** | 3.72 | **0.0** | 4.26 | 2.67 | 5.64 |
| **Pose 3** | 3.31 | 4.26 | **0.0** | 4.84 | 3.87 |
| **Pose 4** | 3.91 | 2.67 | 4.84 | **0.0** | 5.52 |
| **Pose 5** | 5.05 | 5.64 | 3.87 | 5.52 | **0.0** |

f.17a

|  | **Pose 1** | **Pose 2** | **Pose 3** | **Pose 4** | **Pose 5** |
| --- | --- | --- | --- | --- | --- |
| **Pose 1** | **0.0** | 3.80 | 4.31 | 3.18 | 2.08 |
| **Pose 2** | 3.80 | **0.0** | 4.91 | 2.11 | 3.61 |
| **Pose 3** | 4.31 | 4.91 | **0.0** | 4.74 | 3.98 |
| **Pose 4** | 3.18 | 2.11 | 4.74 | **0.0** | 3.48 |
| **Pose 5** | 2.08 | 3.61 | 3.98 | 3.48 | **0.0** |

|  | **Pose 1** | **Pose 2** | **Pose 3** | **Pose 4** | **Pose 5** |
| --- | --- | --- | --- | --- | --- |
| **Pose 1** | **0.0** | 5.57 | 5.56 | 8.53 | 5.04 |
| **Pose 2** | 5.57 | **0.0** | 3.37 | 5.00 | 6.49 |
| **Pose 3** | 5.56 | 3.37 | **0.0** | 6.56 | 5.82 |
| **Pose 4** | 8.53 | 5.00 | 6.56 | **0.0** | 8.55 |
| **Pose 5** | 5.04 | 6.49 | 5.82 | 8.55 | **0.0** |

g. Pilsicainide

|  | **Pose 1** | **Pose 2** | **Pose 3** | **Pose 4** | **Pose 5** |
| --- | --- | --- | --- | --- | --- |
| **Pose 1** | **0.0** | 3.78 | 2.33 | 4.05 | 2.74 |
| **Pose 2** | 3.78 | **0.0** | 2.66 | 3.31 | 4.14 |
| **Pose 3** | 2.33 | 2.66 | **0.0** | 3.86 | 3.74 |
| **Pose 4** | 4.05 | 3.31 | 3.86 | **0.0** | 2.99 |
| **Pose 5** | 2.74 | 4.14 | 3.74 | 2.99 | **0.0** |

h. Mepivacaine

|  | **Pose 1** | **Pose 2** | **Pose 3** | **Pose 4** | **Pose 5** |
| --- | --- | --- | --- | --- | --- |
| **Pose 1** | **0.0** | 4.33 | 6.24 | 3.58 | 3.93 |
| **Pose 2** | 4.33 | **0.0** | 4.99 | 3.99 | 3.40 |
| **Pose 3** | 6.24 | 4.99 | **0.0** | 5.81 | 5.63 |
| **Pose 4** | 3.58 | 3.99 | 5.81 | **0.0** | 2.51 |
| **Pose 5** | 3.93 | 3.40 | 5.63 | 2.51 | **0.0** |

|  | **Pose 1** | **Pose 2** | **Pose 3** | **Pose 4** | **Pose 5** |
| --- | --- | --- | --- | --- | --- |
| **Pose 1** | **0.0** | 3.39 | 5.60 | 3.93 | 4.72 |
| **Pose 2** | 3.39 | **0.0** | 7.50 | 5.64 | 6.17 |
| **Pose 3** | 5.60 | 7.50 | **0.0** | 6.20 | 5.45 |
| **Pose 4** | 3.93 | 5.64 | 6.20 | **0.0** | 4.11 |
| **Pose 5** | 4.72 | 6.17 | 5.45 | 4.11 | **0.0** |

**Supplementary Table 2: MMGBSA binding free energies (kcal/mol)**

| **Drug** | **Cisapride** | **Mosapride** | **13a** | **17a** | **Ranolazine** | **Lidocaine** | **Pilsicainide** | **Mepivacaine** |
| --- | --- | --- | --- | --- | --- | --- | --- | --- |
| **Pose 1** | -40.15±3.25 | -38.32±2.54 | **-34.18±4.03** | -26.03±7.43 | -26.68±2.31 | -19.27±2.68 | -23.22±3.16 | **-22.14±3.63** |
| **Pose 2** | -35.65±5.30 | -29.63±2.75 | -19.06±3.03 | **-37.57±5.16** | -30.11±3.01 | -17.57±3.70 | **-28.71±3.90** | -19.72±3.01 |
| **Pose 3** | **-44.95±3.68** | -37.25±3.76 | -22.02±2.13 | -18.80±3.51 | -28.12±3.20 | **-26.05±4.68** | -24.82±3.67 | -19.68±2.46 |
| **Pose 4** | -42.33±2.53 | **-39.91±4.07** | -29.63±3.42 | -35.18±4.82 | -30.86±3.25 | -13.17±2.65 | -22.47±3.37 | -18.97±2.79 |
| **Pose 5** | -38.98±5.55 | -25.20±3.27 | -26.89±3.88 | -29.40±3.69 | **-35.15±3.19** | -17.76±2.89 | -15.58±3.24 | -20.85±3.41 |

**Supplementary Table 3: Free energies vs experimental affinities**

| Drug | MMGBSA kcal/mol | Approximate ABF free energy kcal/mol | Literature IC50 (M) |
| --- | --- | --- | --- |
| Cisapride | -44.95 | -72 | 9.4 x 10^-9^ |
| Mosapride | -39.91 | -42 | 4.8 x 10^-6^ |
| Ranolazine | -35.15 | -38 | 8.03 x 10^-6^ |
| 13a | -34.18 | -28 | >10 x 10^-6^ |
| 17a | -37.57 | -30 | >10 x 10^-6^ |
| Pilsicainide | -28.71 | -29 | 20.4 x 10^-6^ |
| Lidocaine | -26.05 | -21 | 141.77 x 10^-6^ |
| Mepivacaine | -22.14 | -21 | 156.2 x 10^-6^ |


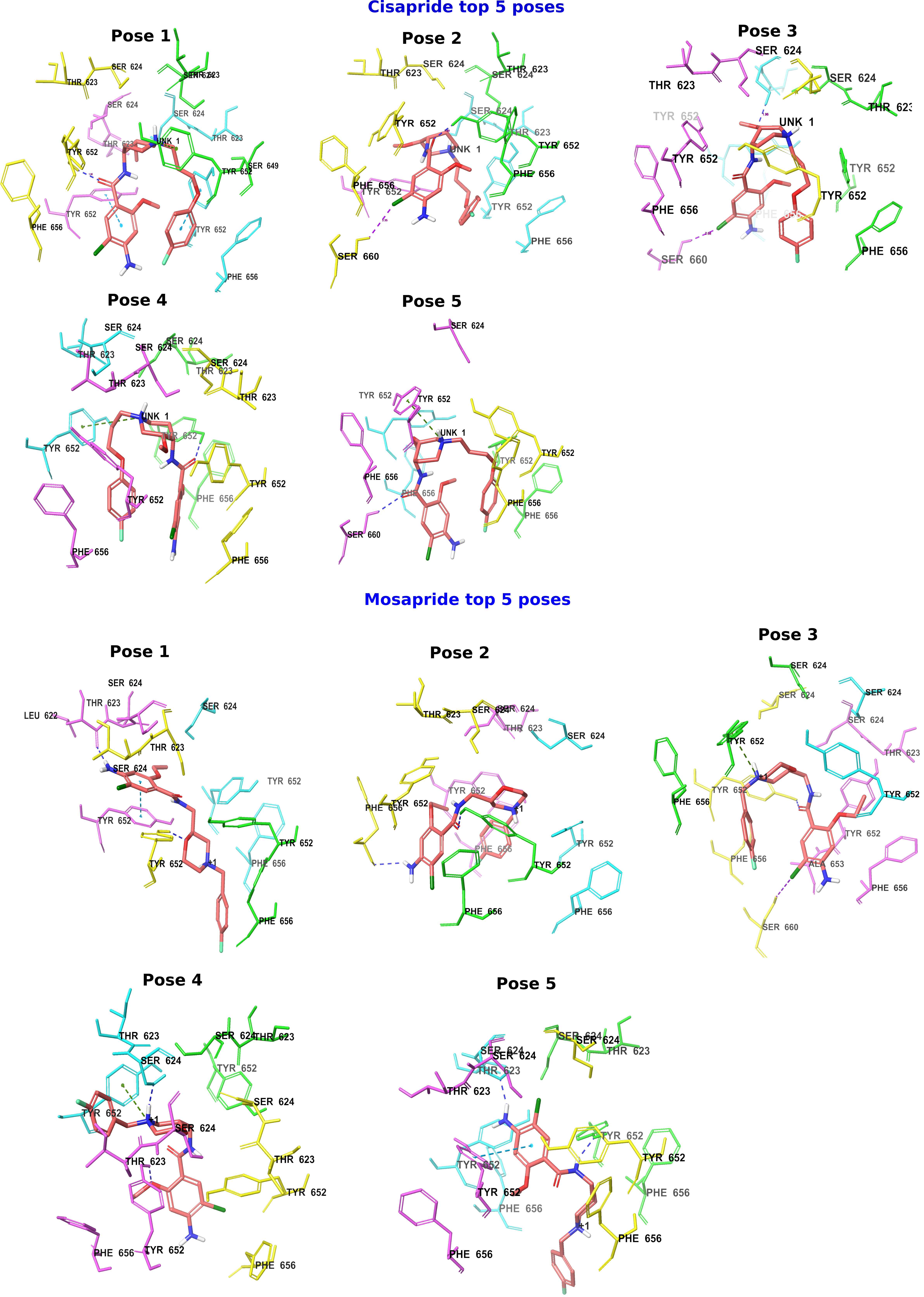


**Supplementary Figure 1:** Best scoring poses identified for Cisapride (top panel) and Mosapride (Bottom panel) from Docking


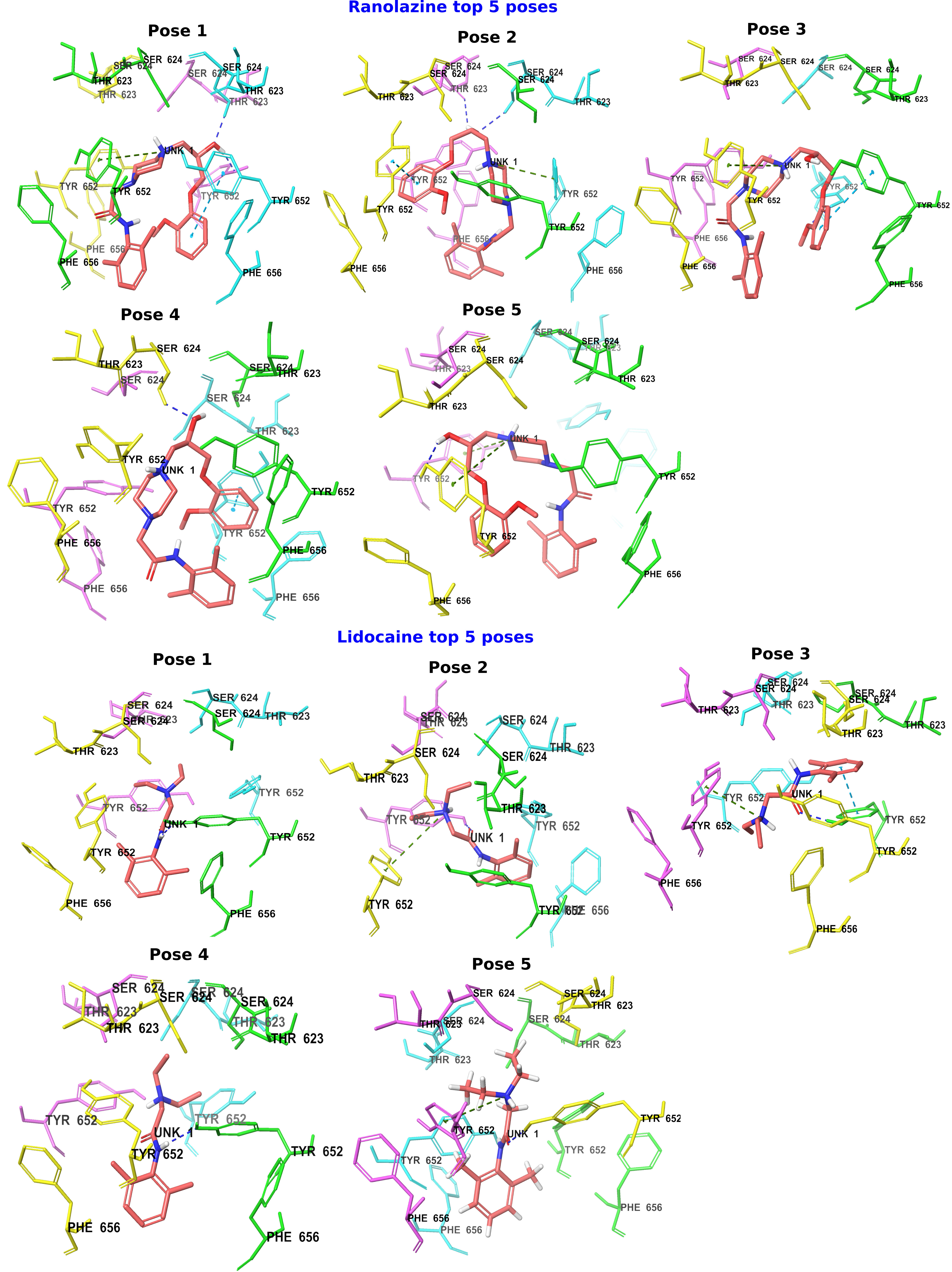


**Supplementary figure 2:** Best scoring poses identified for Ranolazine (top panel) and Lidocaine (Bottom panel) from Docking


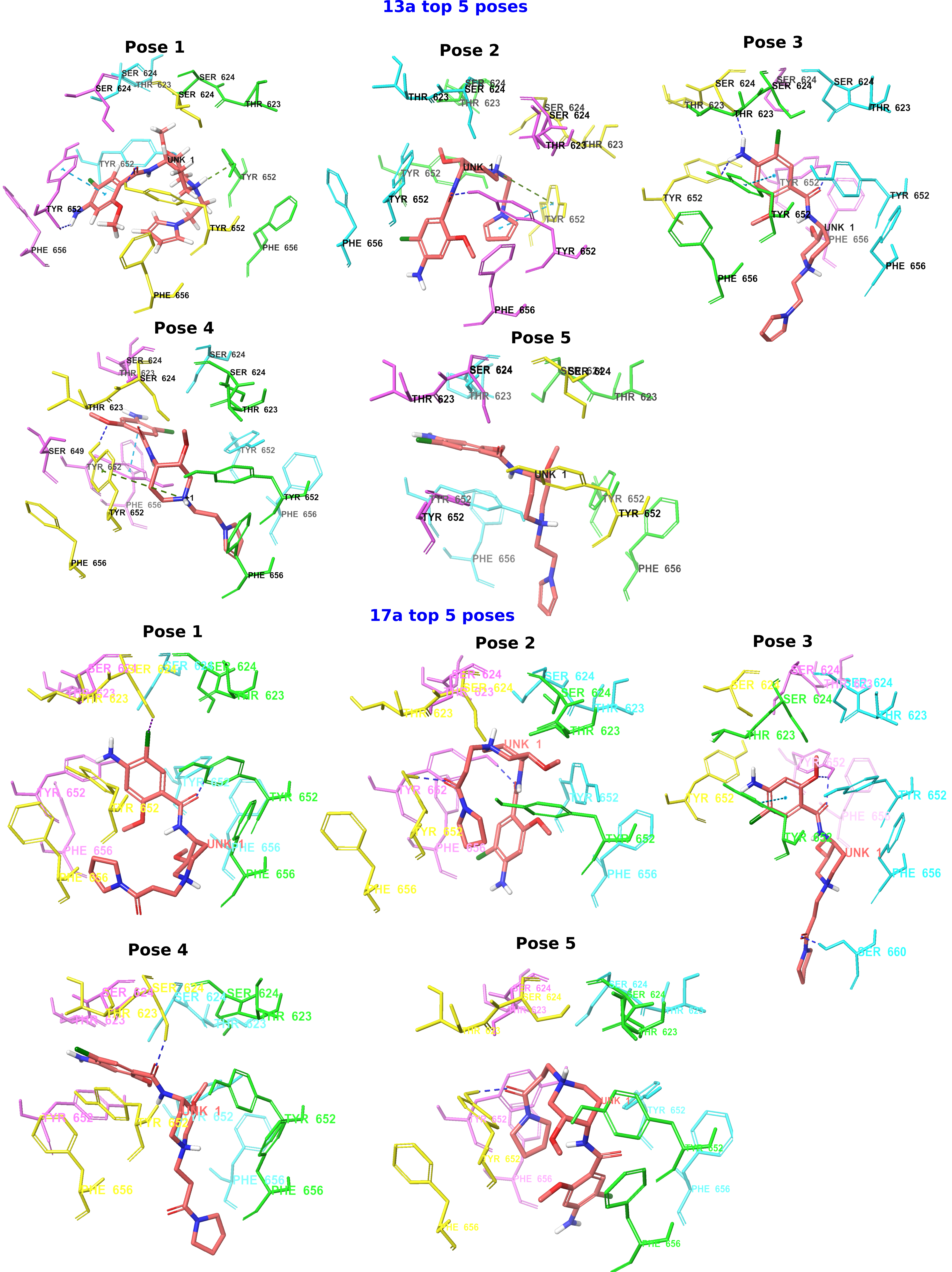


**Supplementary figure 3:** Best scoring poses identified for Cisapride analogs, 13a (top panel) and 17a (Bottom panel) from Docking


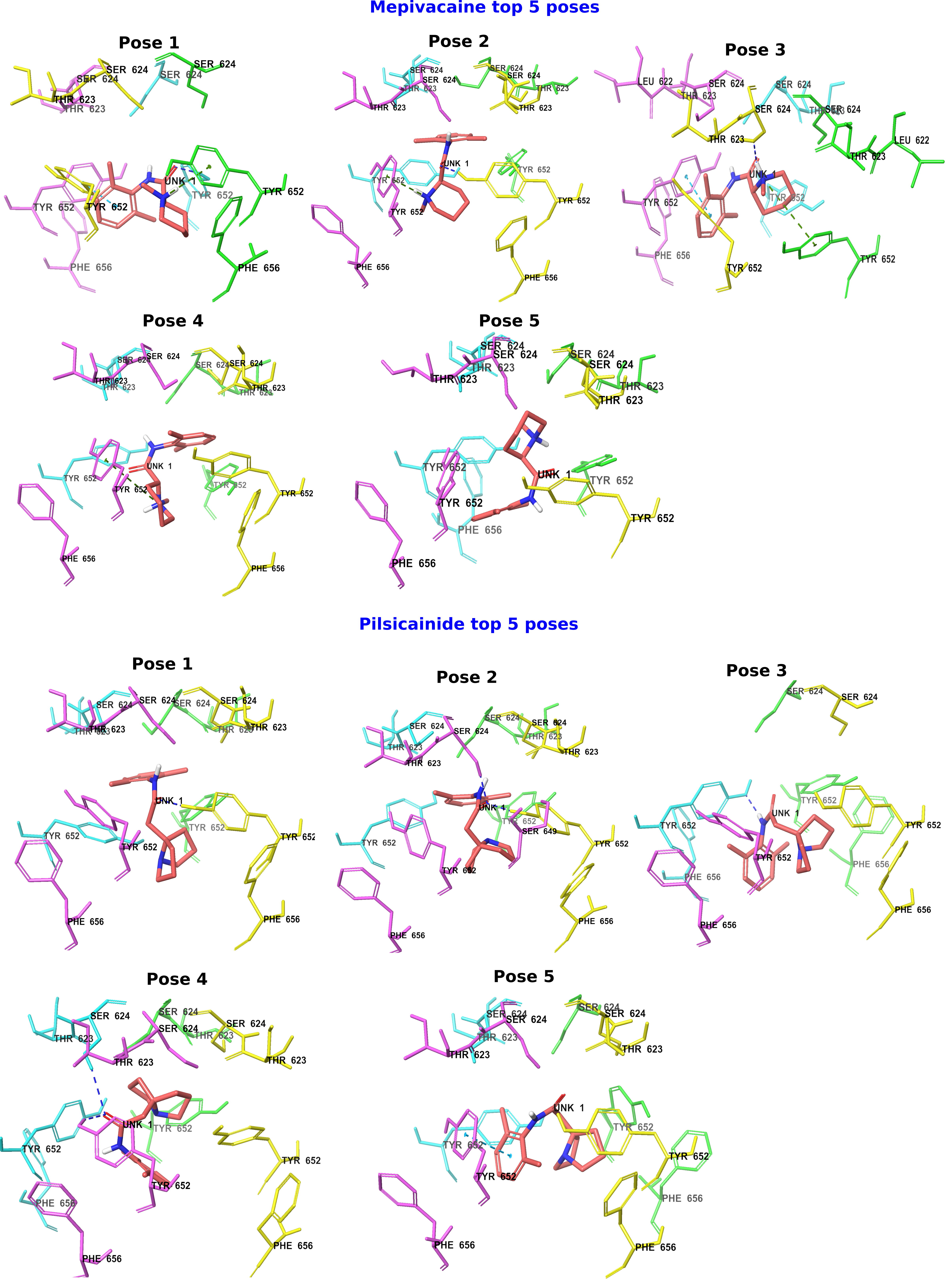


**Supplementary figure 4:** Best scoring poses identified for Cisapride analogs, Pilsicainide (top panel) and Mepivacaine (Bottom panel) from Docking


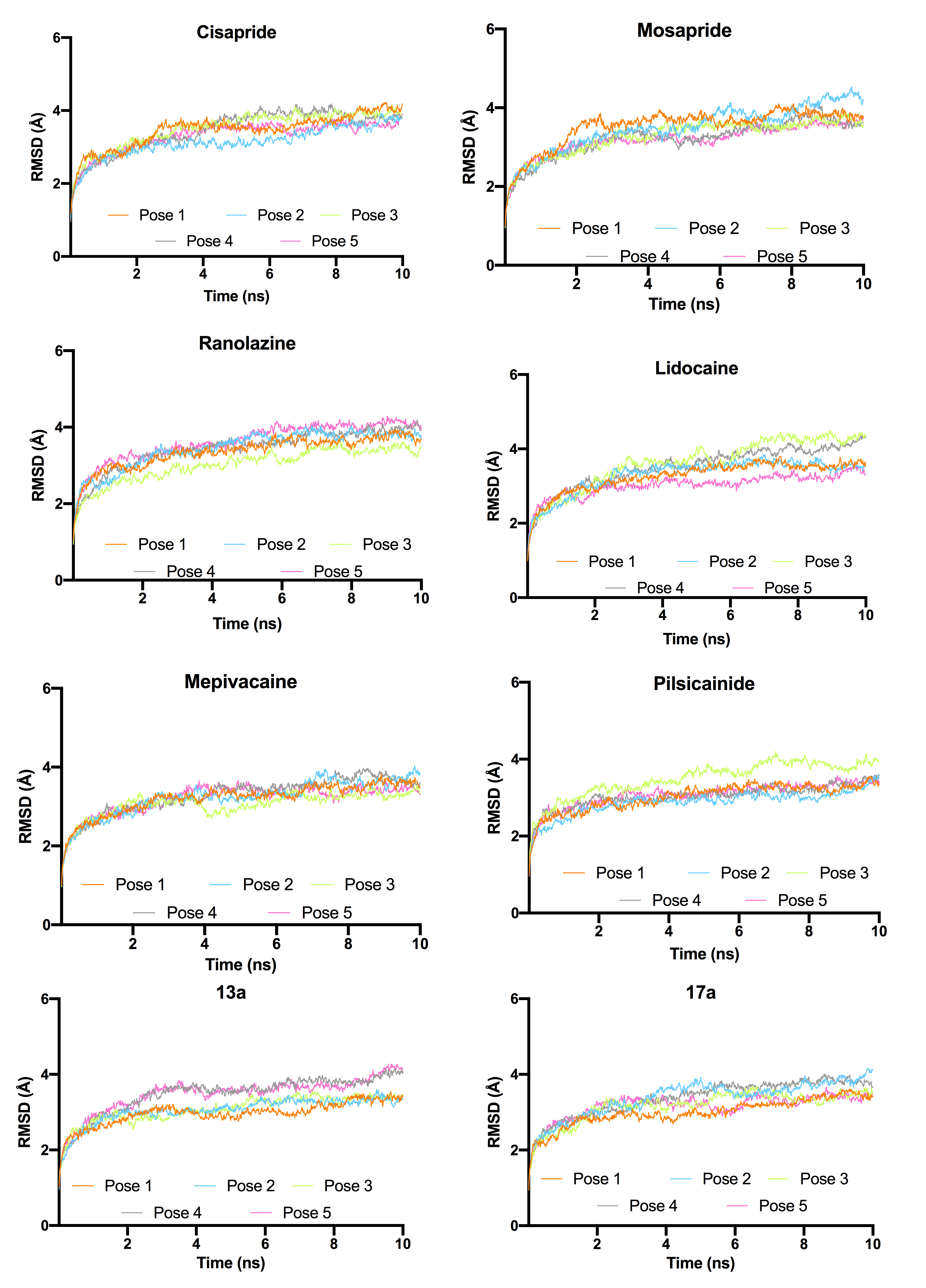


**Supplementary figure 5:** RMSDs of all five poses of the drugs observed in the 10 ns classical MD equilibration protocol


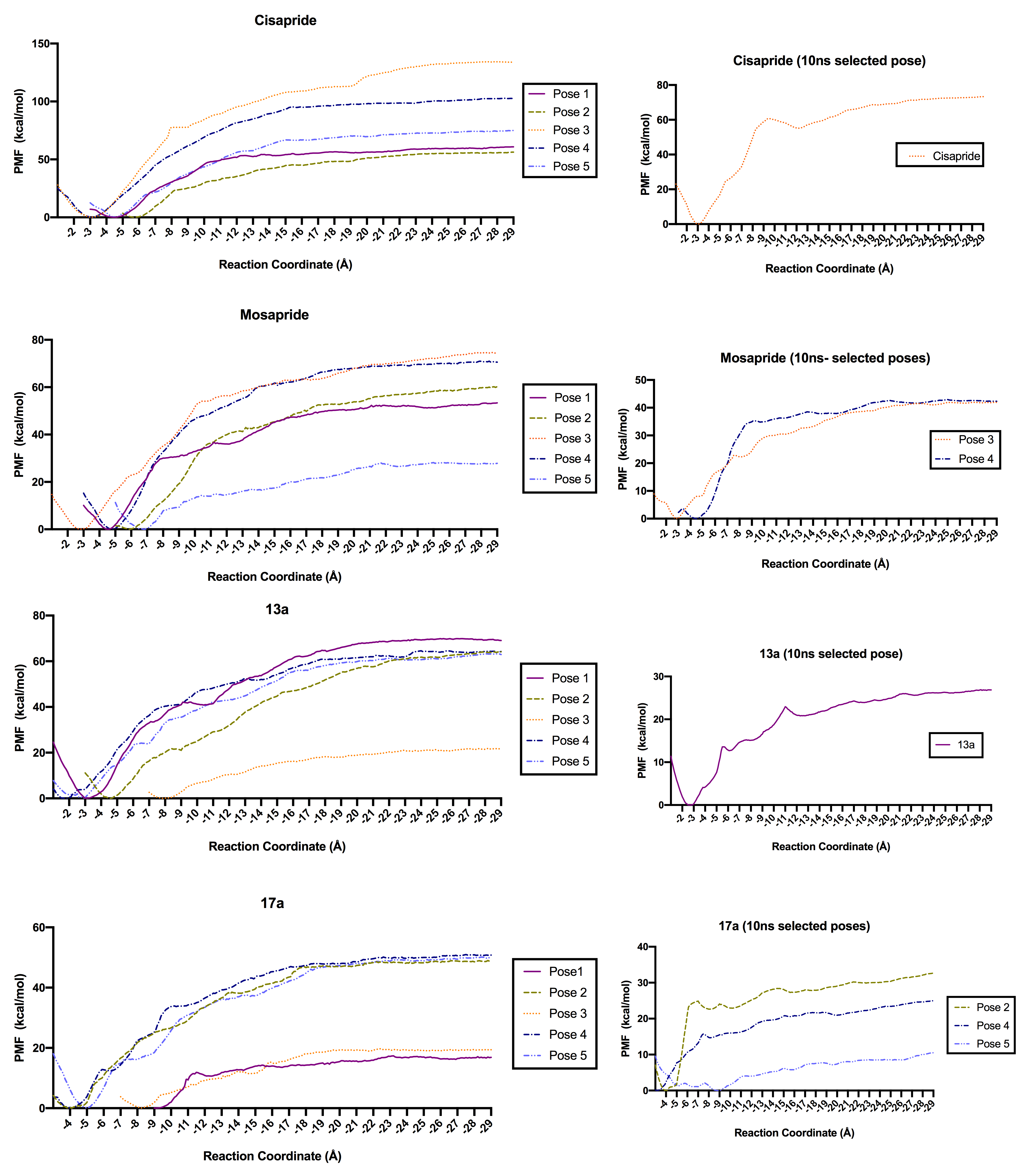


**Supplementary figure 6:** PMF profiles of five poses of Cisapride, Mosapride, 13a and 17a calculated from 1 ns ABF simulations (left panel). PMF profiles of poses showing the best and/or similar range of free energies were simulated using the ABF protocol for an extended timescale of 10 ns (right panel)


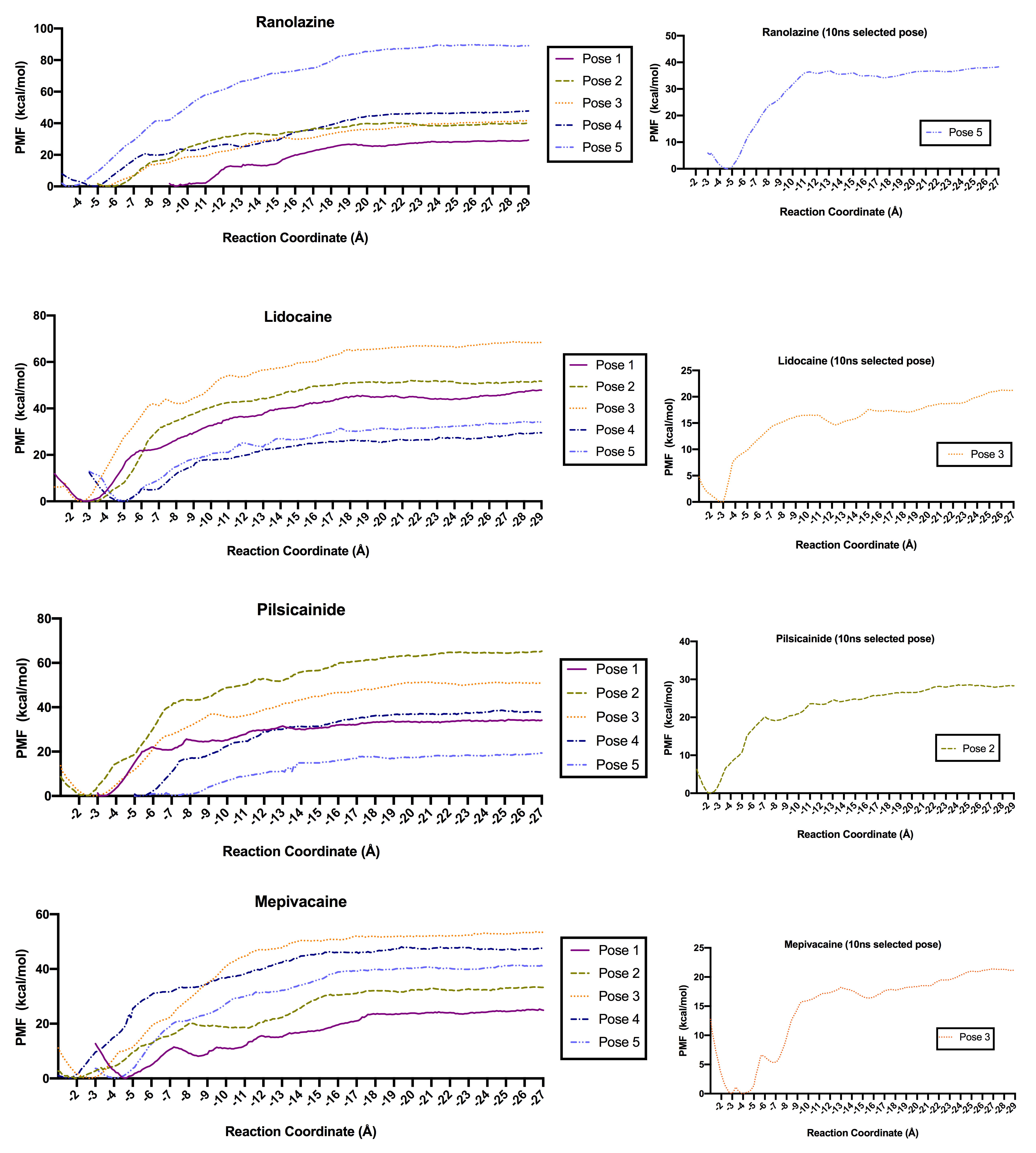


**Supplementary figure 7:** PMF profiles of five poses of Ranolazine, Lidocaine, Pilsicainide and Mepivacaine calculated from 1 ns ABF simulations (left panel). PMF profiles of poses showing the best and/or similar range of free energies were simulated using the ABF protocol for an extended timescale of 10 ns (right panel)


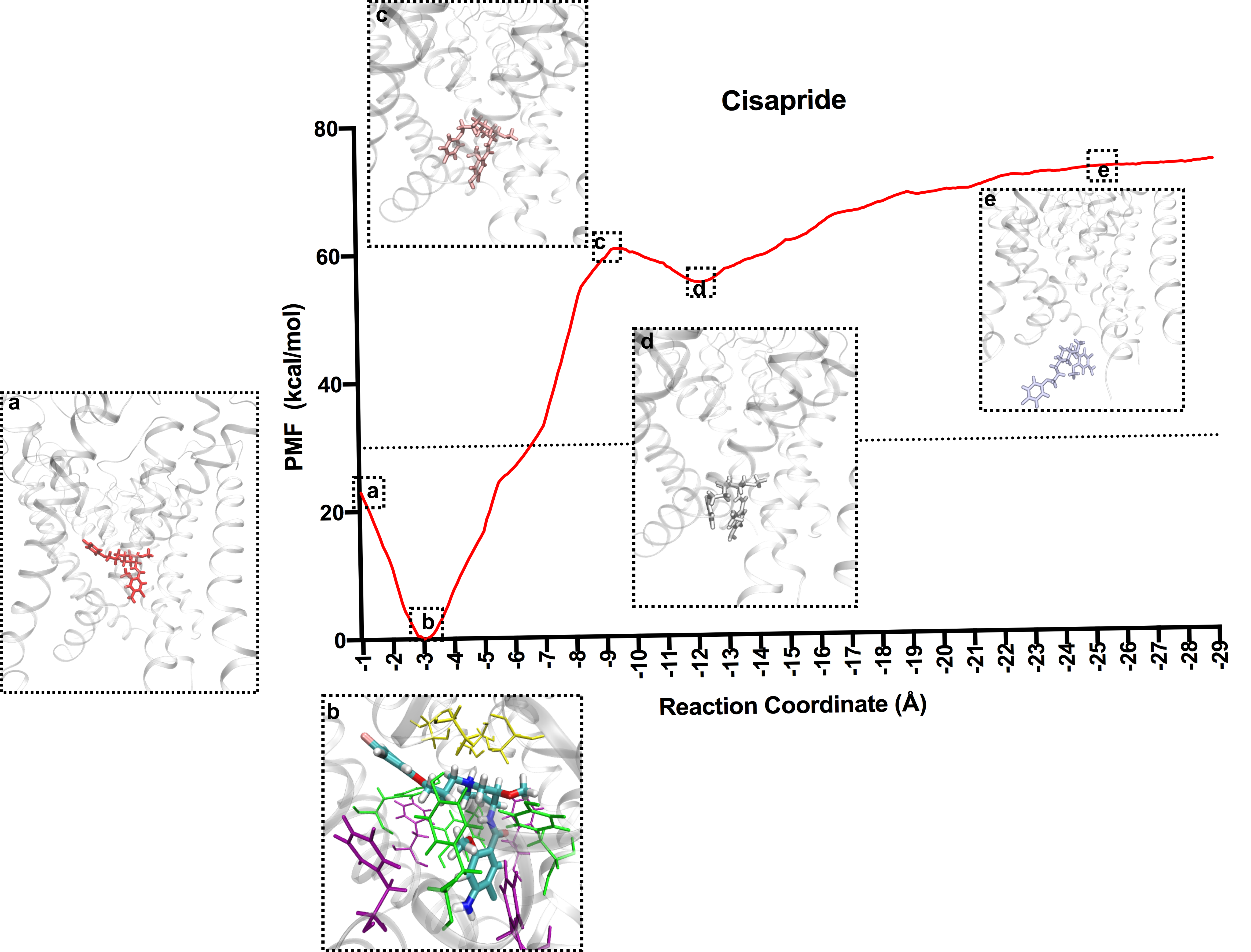


**Supplementary figure 8:** Position of cisapride along the reaction coordinate chosen for the calculation of potential of mean force obtained from the 10 ns ABF simulations. Point ‘a’ refers to the equilibrated complex of cisapride. Point ‘b’ refers to the lowest energy state of cisapride found along the defined reaction coordinate. We propose this point as the likely mode of binding for this molecule. Point ‘c’ at -9 Å, shows that cisapride moved towards its sub-optimal conformation (as the one similar to the initial docked pose). Point ‘d’, at -12 Å, shows the this suboptimal conformation reaches a local minima by forming intramolecular stacking. At point e, cisapride is located at the intracellular gate.


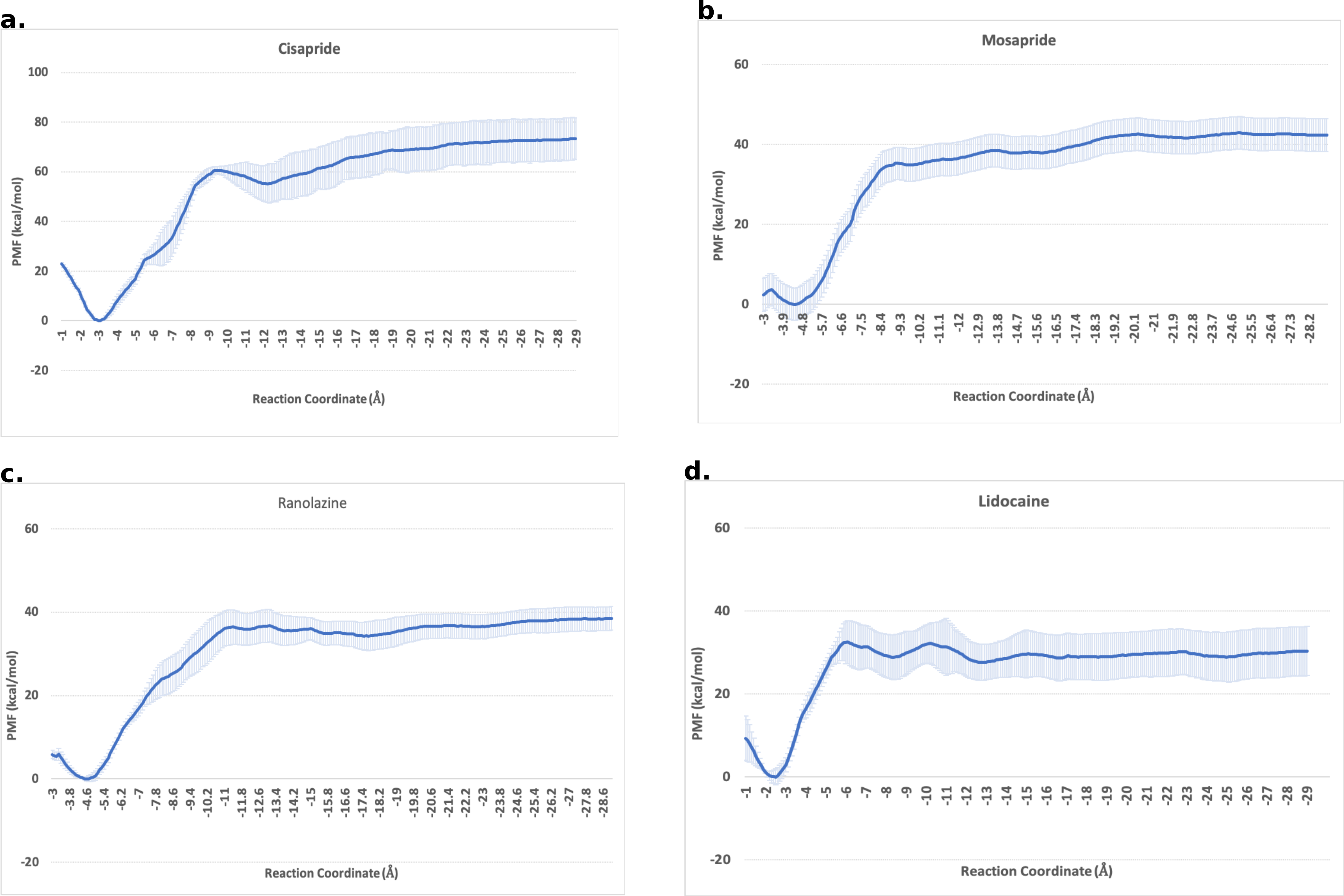


**Supplementary figure9:** Standard deviations to the PMF profiles of Cisapride (a), Mosapride(b), Ranolazine(c), and Lidocaine(d) calculated from 3 repeats of 10 ns ABF simulations.


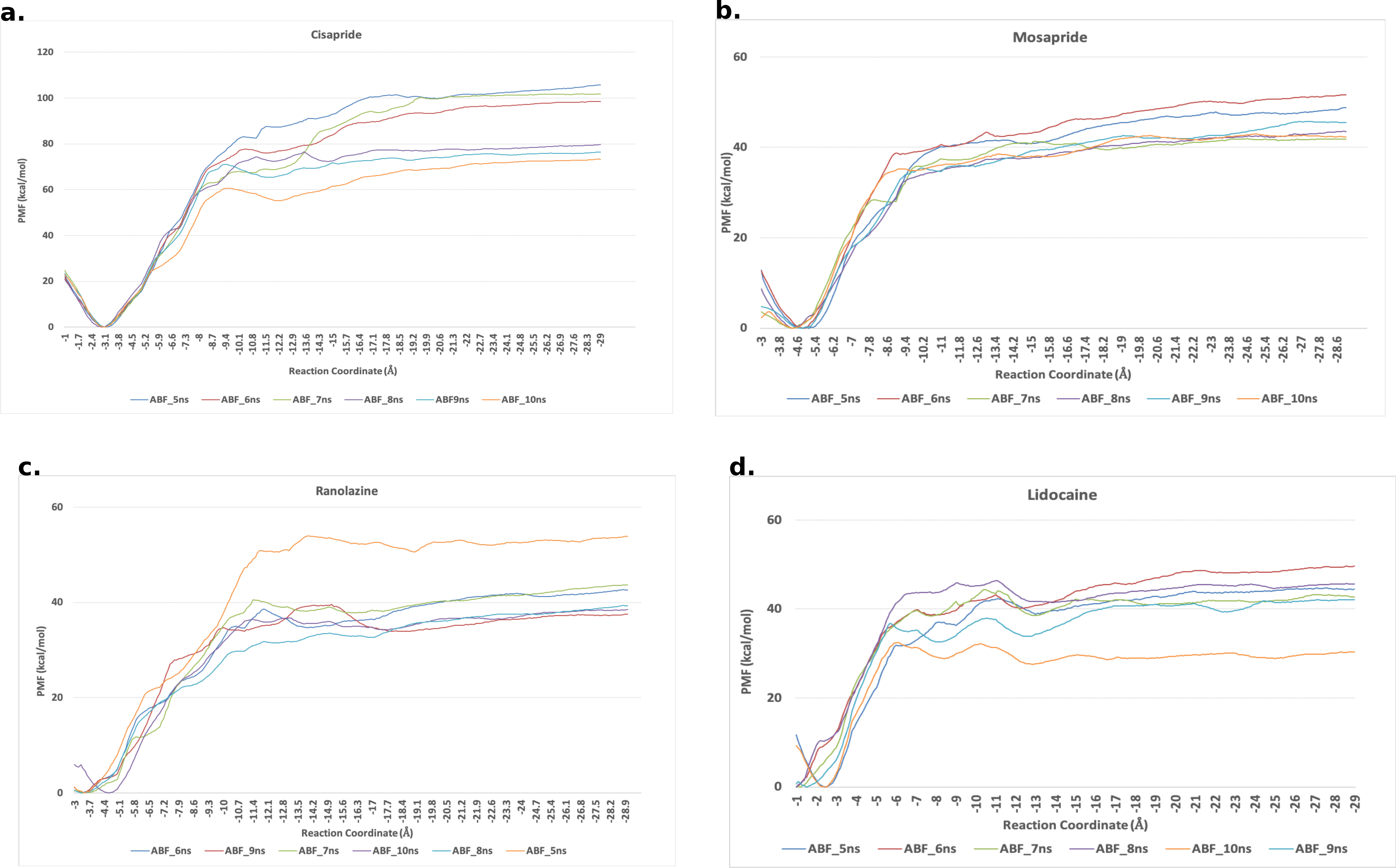


**Supplementary figure 10:** PMF profiles calculated from 5ns, 6ns, 7ns, 8ns,9ns, and 10ns simulation time for each window is shown for Cisapride (a), Mosapride (b), Ranolazine (c), and Lidocaine (d).


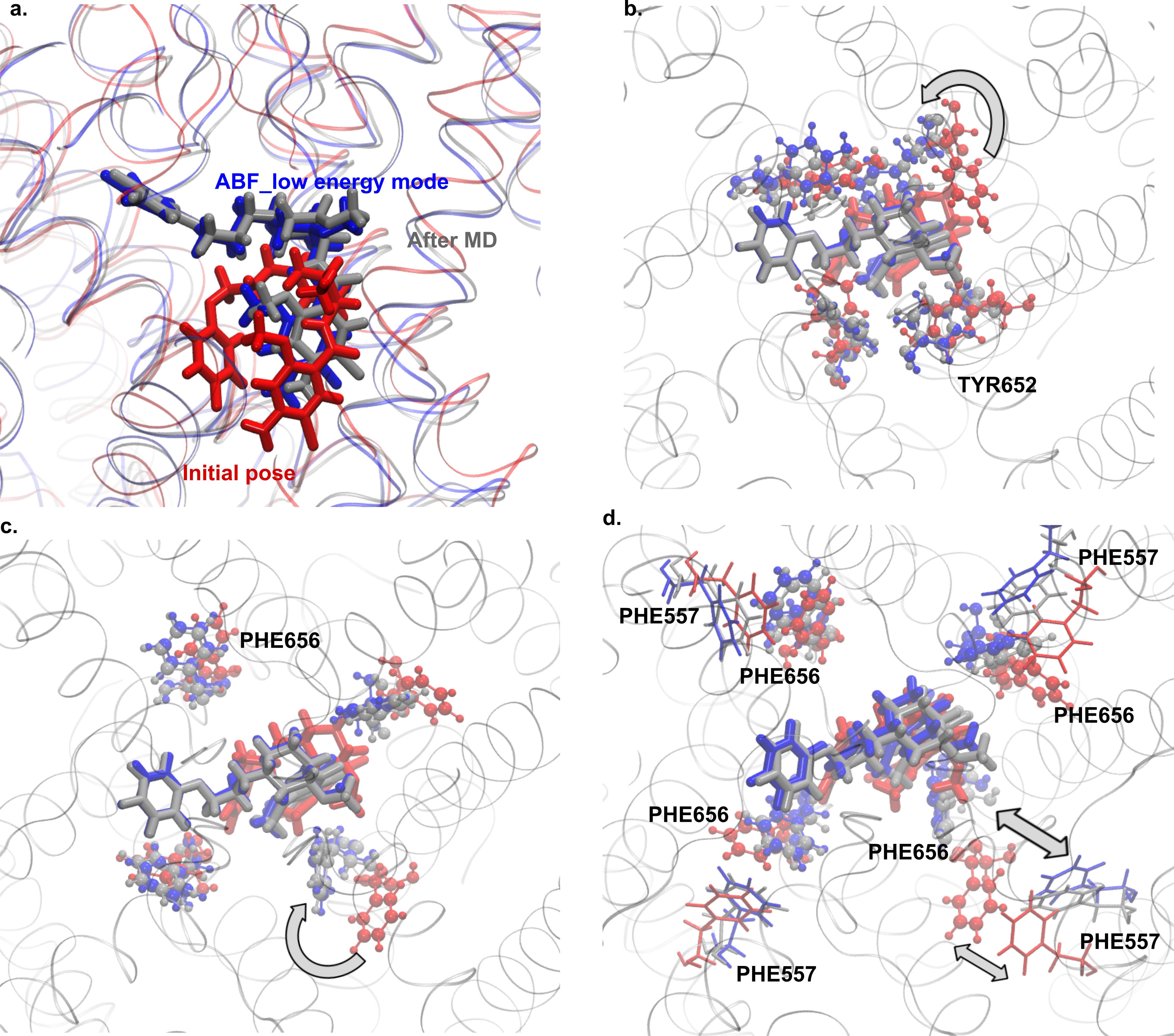


**Supplementary figure 11:** Superimposed structures of cisapride- hERG channel complex (a) initial docked pose (red), post-MD pose (grey), and the low-energy mode identified from ABF(blue) of cisapride as viewed with the extracellular side on the top; (b) Extracellular view of residue orientations on the three structures, Tyr652 (b), Phe656(c), and Phe656 with Phe557(thin sticks)(d)

**
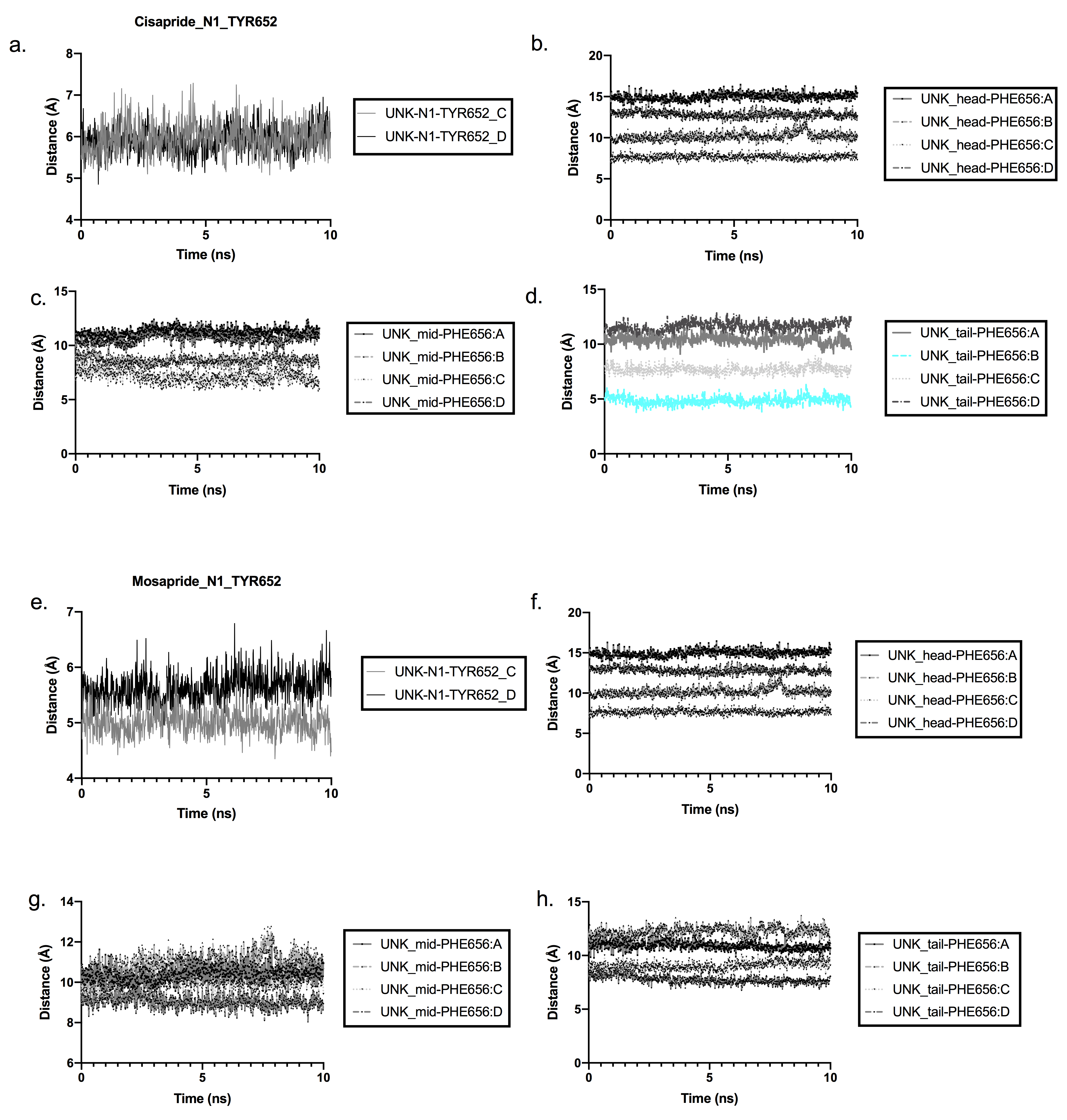
**

**Supplementary Figure 12:** Distance analysis on the cisapride-hERG channel (a-d) and Mosapride-hERG channel complex from 10 ns classical MD simulations (e-h). Distance (in Å) between the Tyr652 rings and the protonated nitrogen of cisapride(a) and mosapride (e). Distance (in Å ) between the center of the head, mid, and tail rings and the PHE656 residue of cisapride (b,c,d) and mosapride (f,g,h). The tail ring interactions of cisapride and PHE656 residue is highlighted with cyan lines in d.


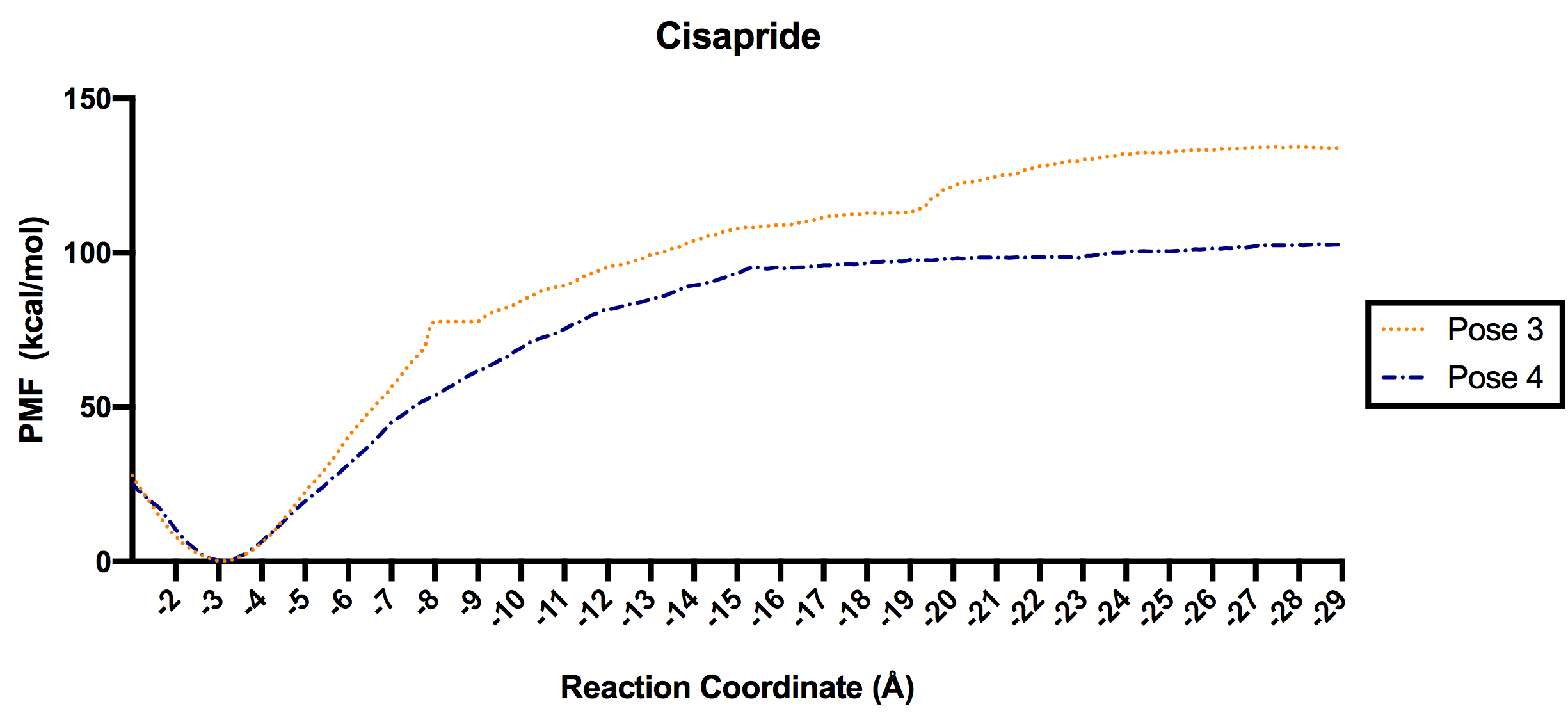

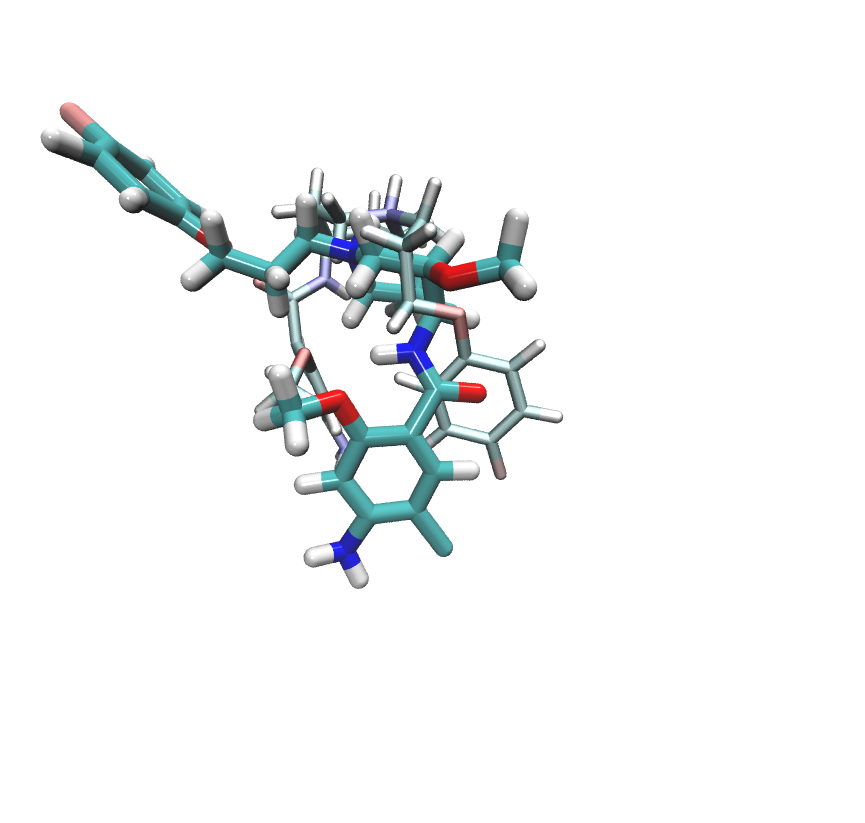


**Supplementary figure 13:** PMF profiles of pose 3 and pose 4 of cisapride calculated from 1 ns ABF simulations (left panel). Starting conformations of pose 3 (thick sticks) and pose4 (thin metallic sticks) as seen with the extracellular side on the top. The head ring in pose 3 occupies the lateral hydrophobic pocket.


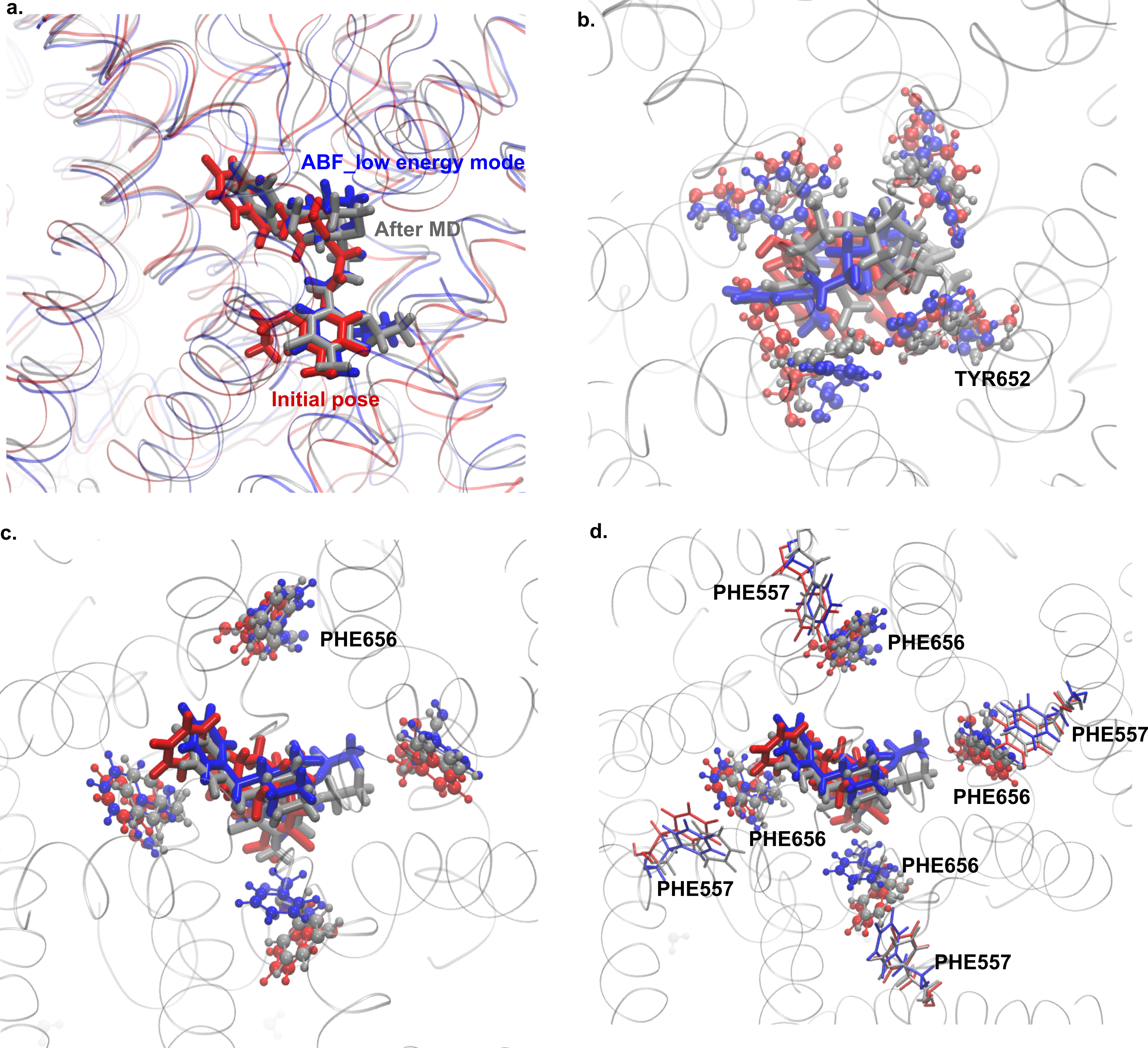


**Supplementary figure 14:** Superimposed structures of mosapride- hERG channel complex (a) initial docked pose (red), post-MD pose (grey), and the low-energy mode identified from ABF(blue) of mosapride as viewed with the extracellular side on the top; (b) Extracellular view of residue orientations on the three structures, Tyr652 (b), Phe656(c), and Phe656 with Phe557(thin sticks)(d)


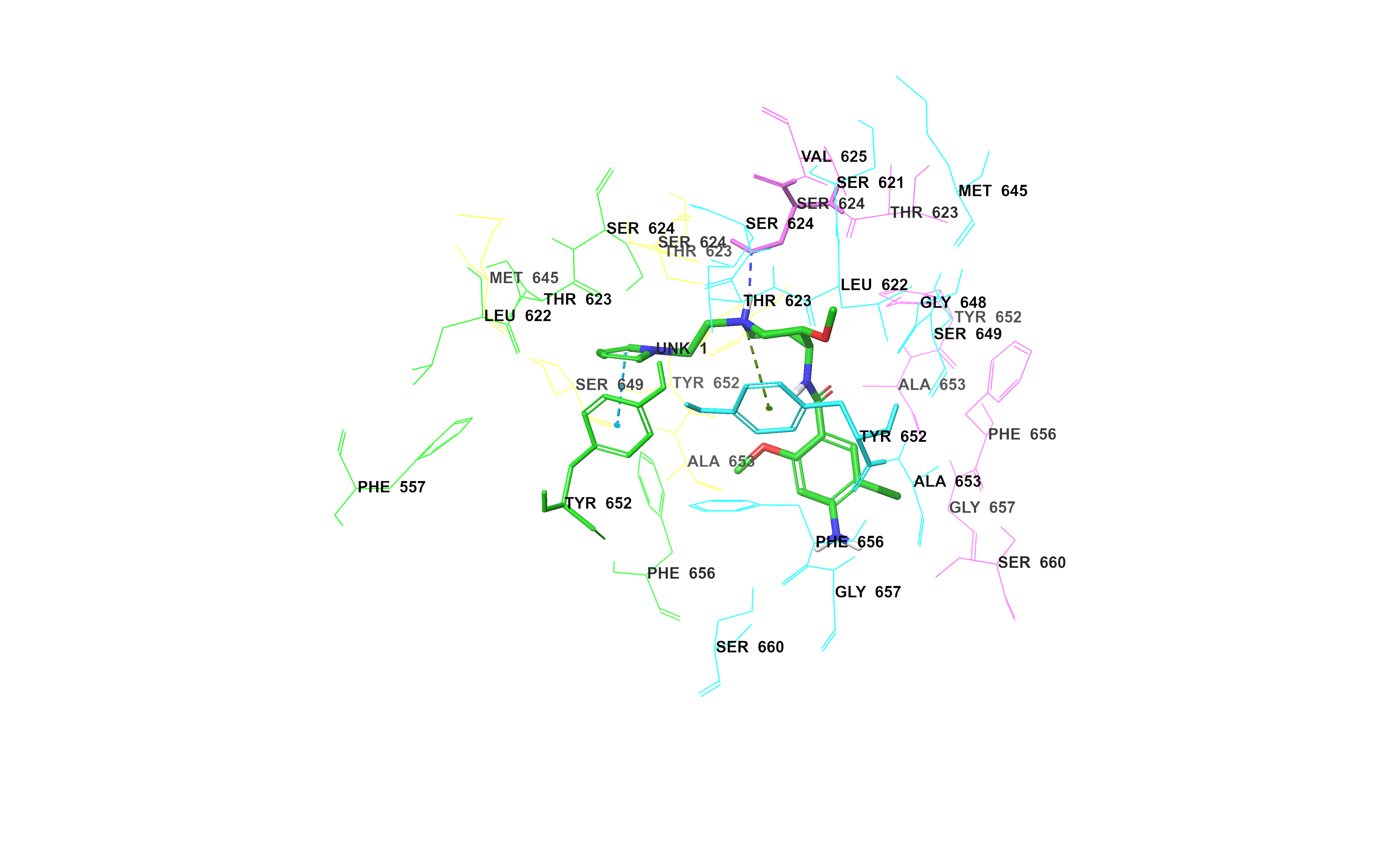

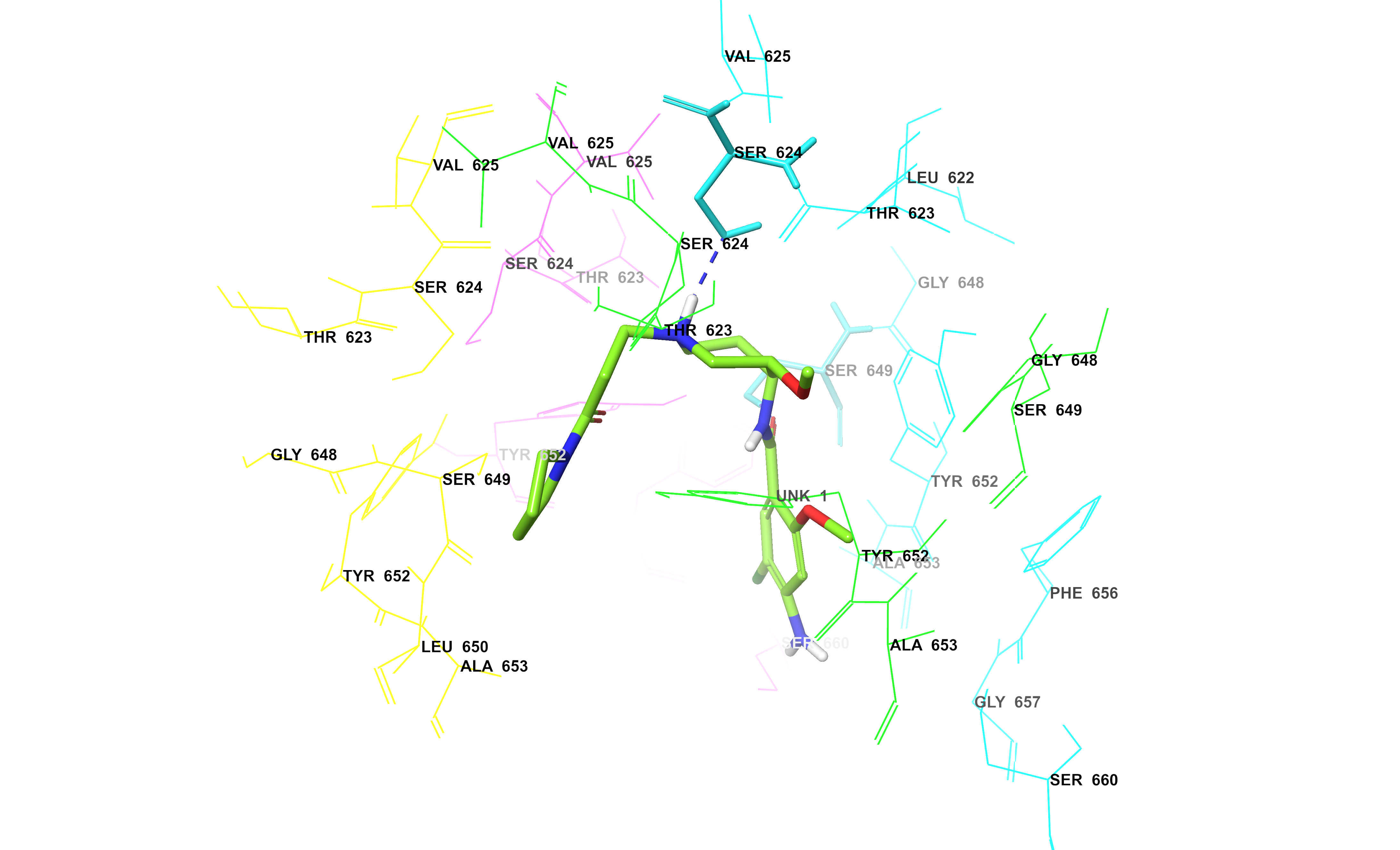


b.

a.

**Supplementary figure 15:** Low-energy binding mode of cisapride analogs, 13a (a) and 17a (b) identified using from the 10 ns ABF simulations as viewed with the extracellular side on the top.


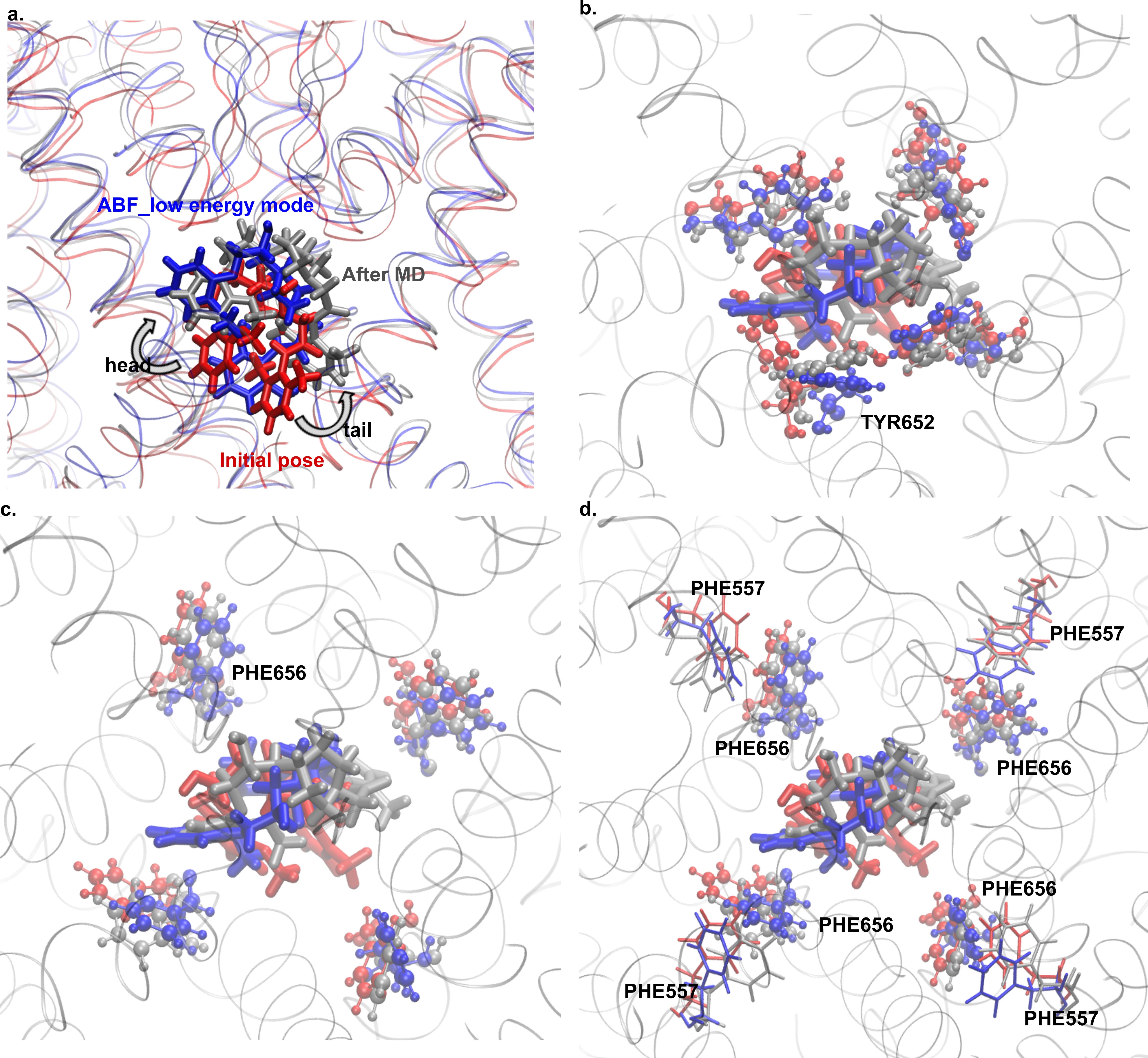


**Supplementary figure 16:** Superimposed structures of ranolazine- hERG channel complex (a) initial docked pose (red), post-MD pose (grey), and the low-energy mode identified from ABF(blue) of ranolazine as viewed with the extracellular side on the top; (b) Extracellular view of residue orientations on the three structures, Tyr652 (b), Phe656(c), and Phe656 with Phe557-thin sticks (d)


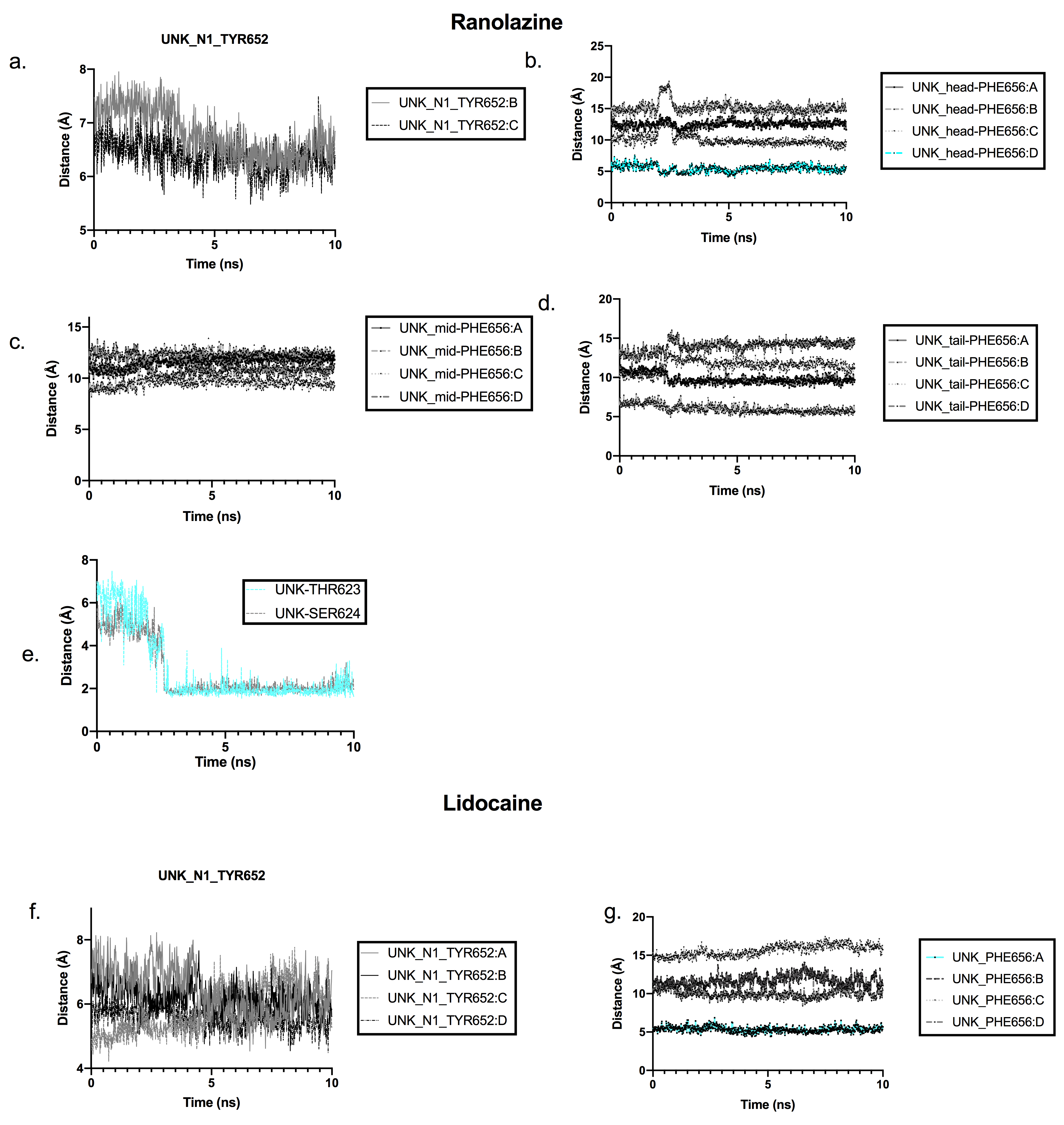


**Supplementary figure 17:** Distance analysis on the ranolazine-hERG channel (a-e) and lidocaine-hERG channel from 10 ns classical MD simulations (f-g). Distance (in Å) between the Tyr652 rings and the protonated nitrogen of ranolazine(a) and lidocaine (f). Distance (in Å ) between the center of the head, mid, and tail rings of ranolazine (b,c,d) and the PHE656 residue; Distance between ranolazine and THR623 and SER624 residues (e); Distance (in Å ) between the ring in lidocaine and PHE656 residue (g). The head ring interactions of ranolzaine and PHE656 residue is highlighted with cyan lines(b)


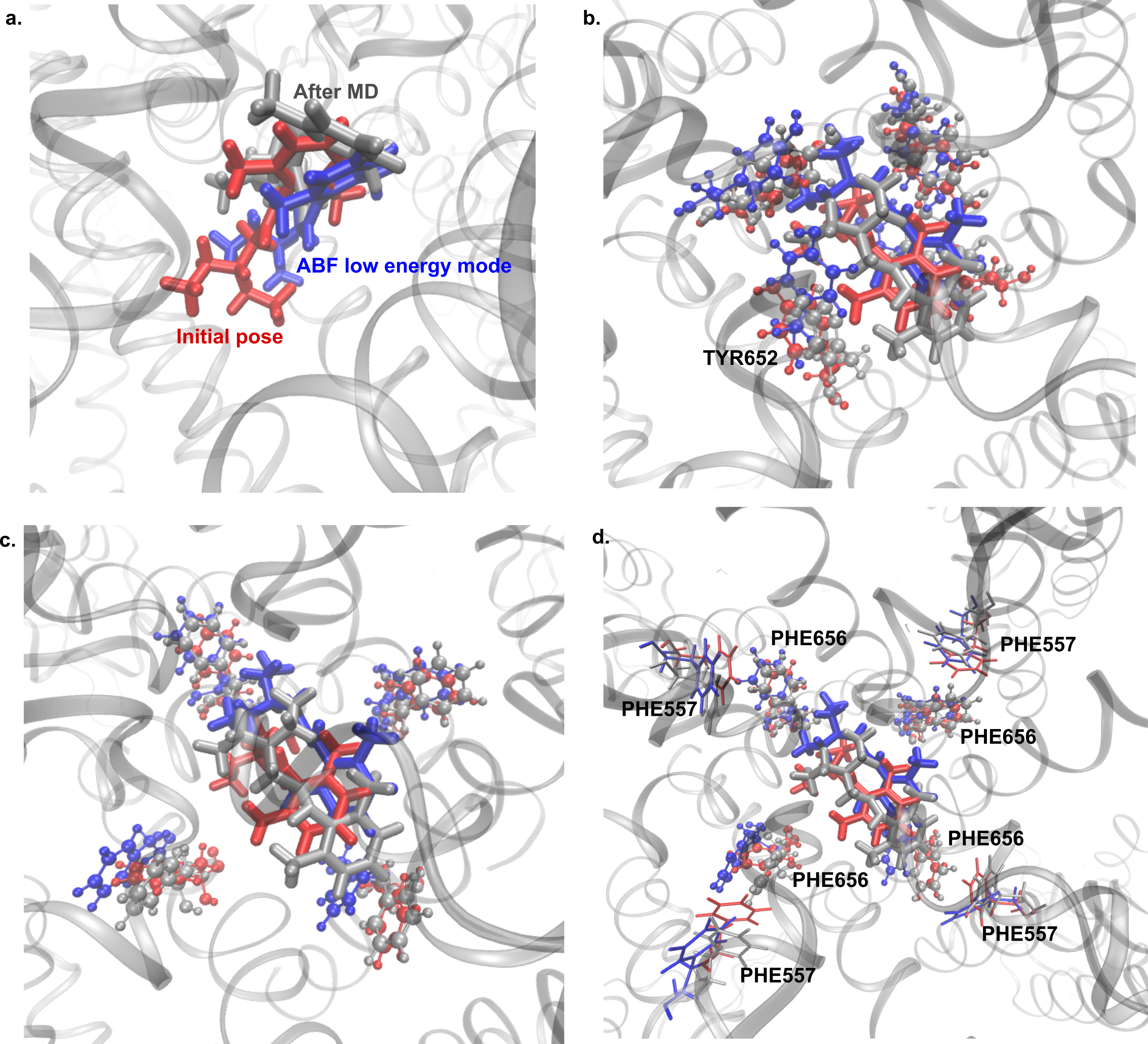


**Supplementary figure 18:** Superimposed structures of Lidocaine- hERG channel complex (a) initial docked pose (red), post-MD pose (grey), and the low-energy mode identified from ABF(blue) of lidocaine as viewed with the extracellular side on the top; (b) Extracellular view of residue orientations on the three structures, Tyr652 (b), Phe656(c), and Phe656 with Phe557-thin sticks (d)


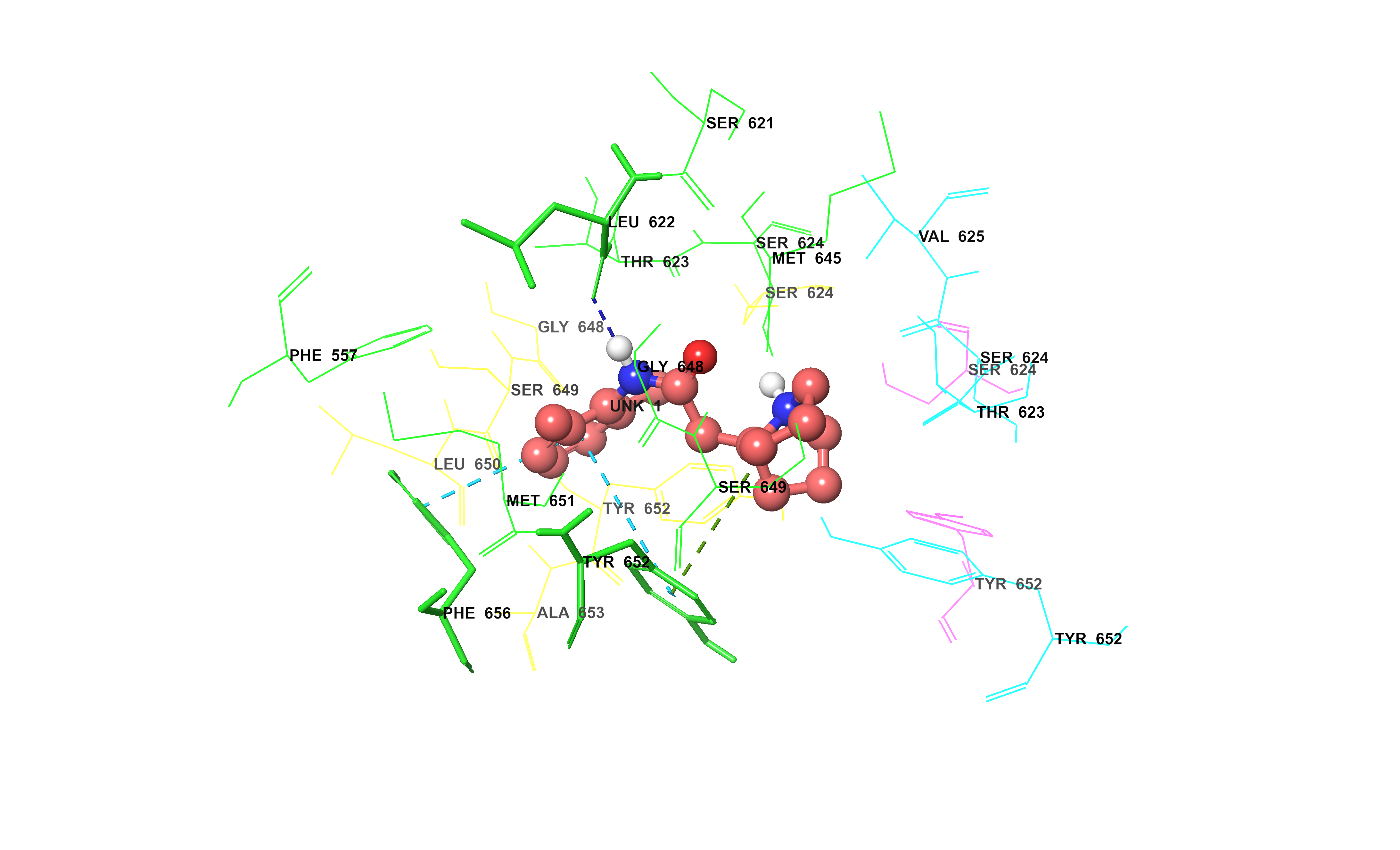

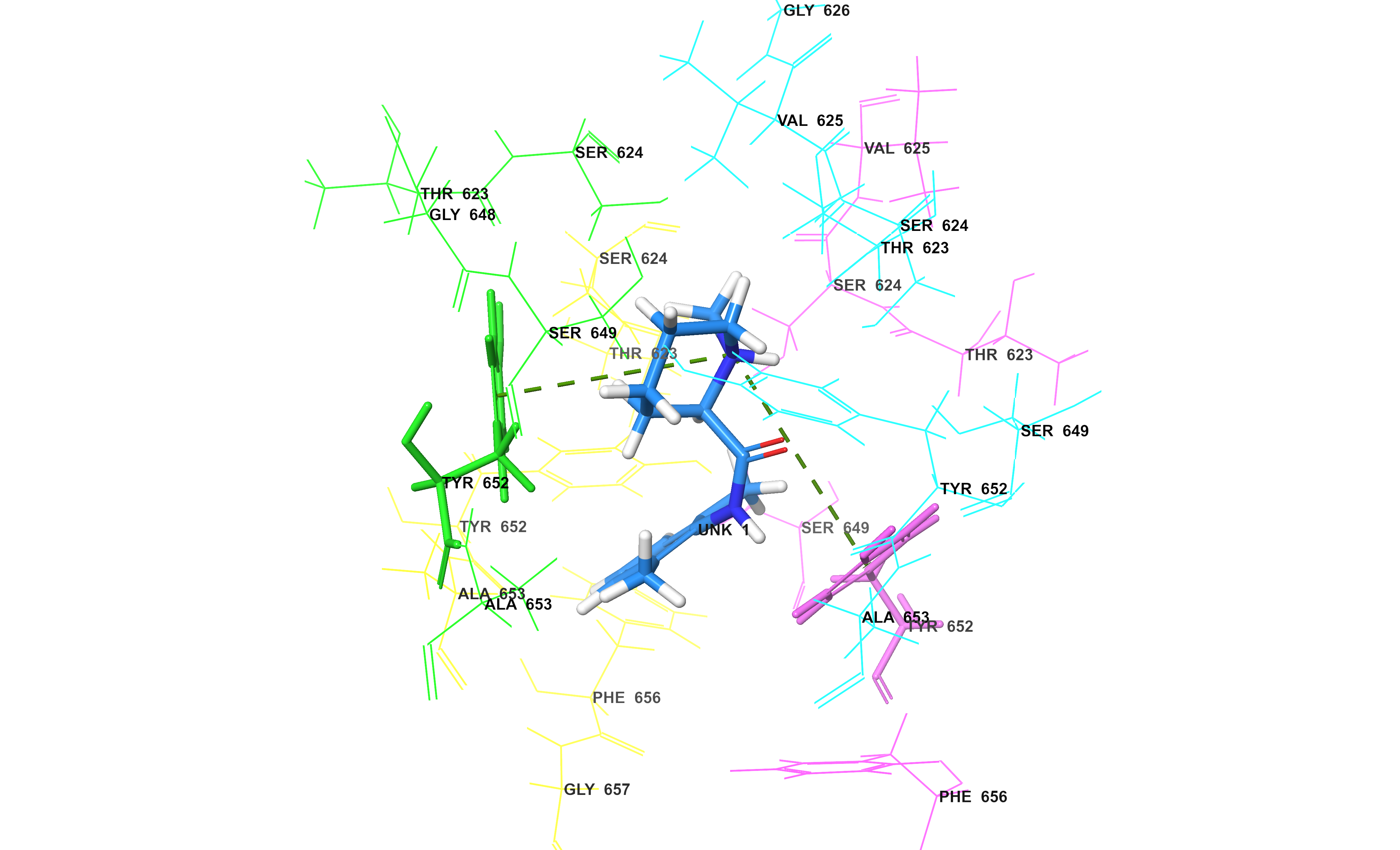


b.

a.

**Supplementary Figure 19:** Low-energy binding mode of Pilsicainide (a) and Mepivacaine (b) identified using from the 10 ns ABF simulations as viewed with the extracellular side on the top.


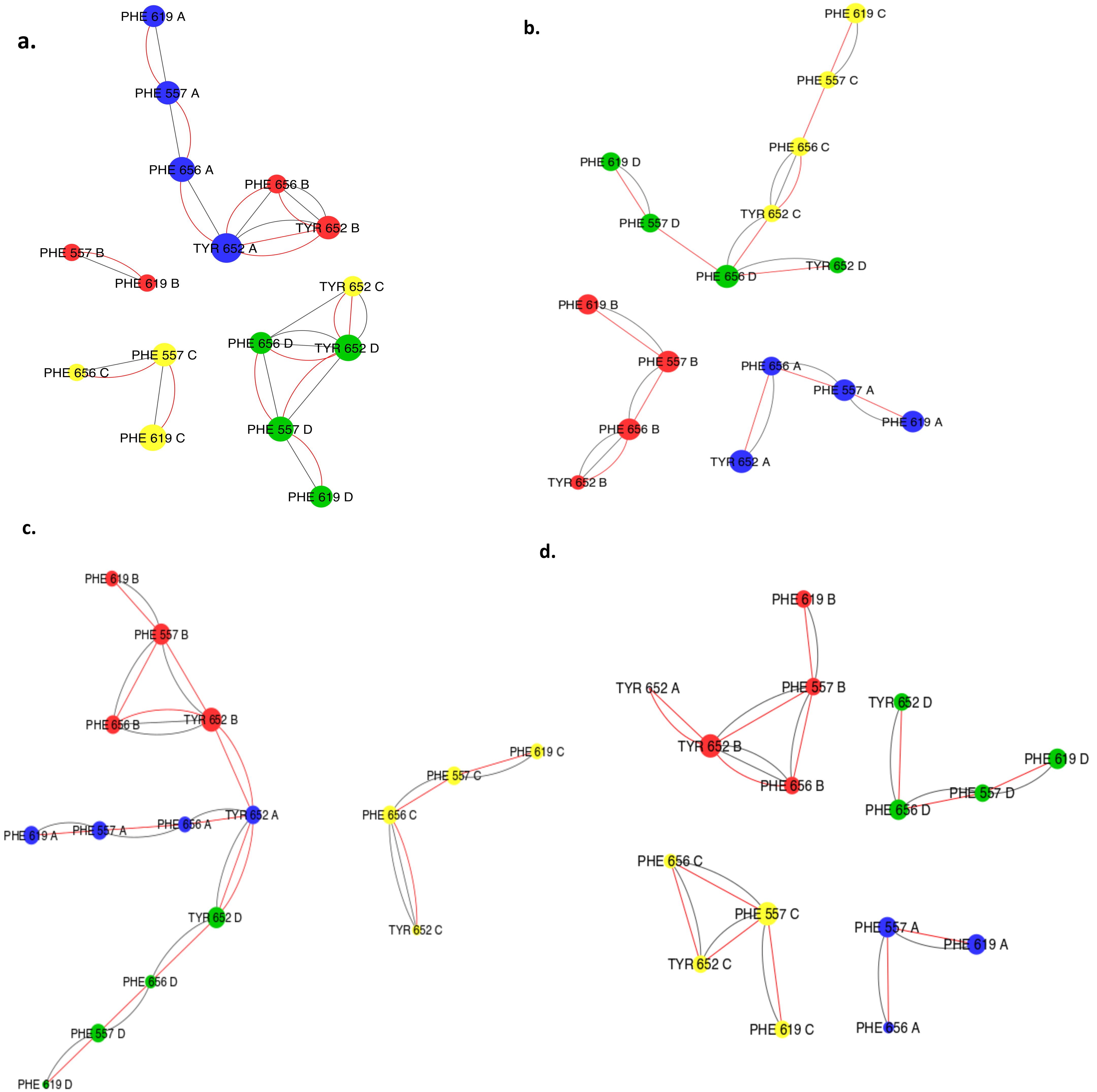


**Supplementary Figure 20:** Pi-Pi network observed from the low-energy complex structures. (a) Cisapride (b) Mosapride (c) Ranolazine (d) Lidocaine

**
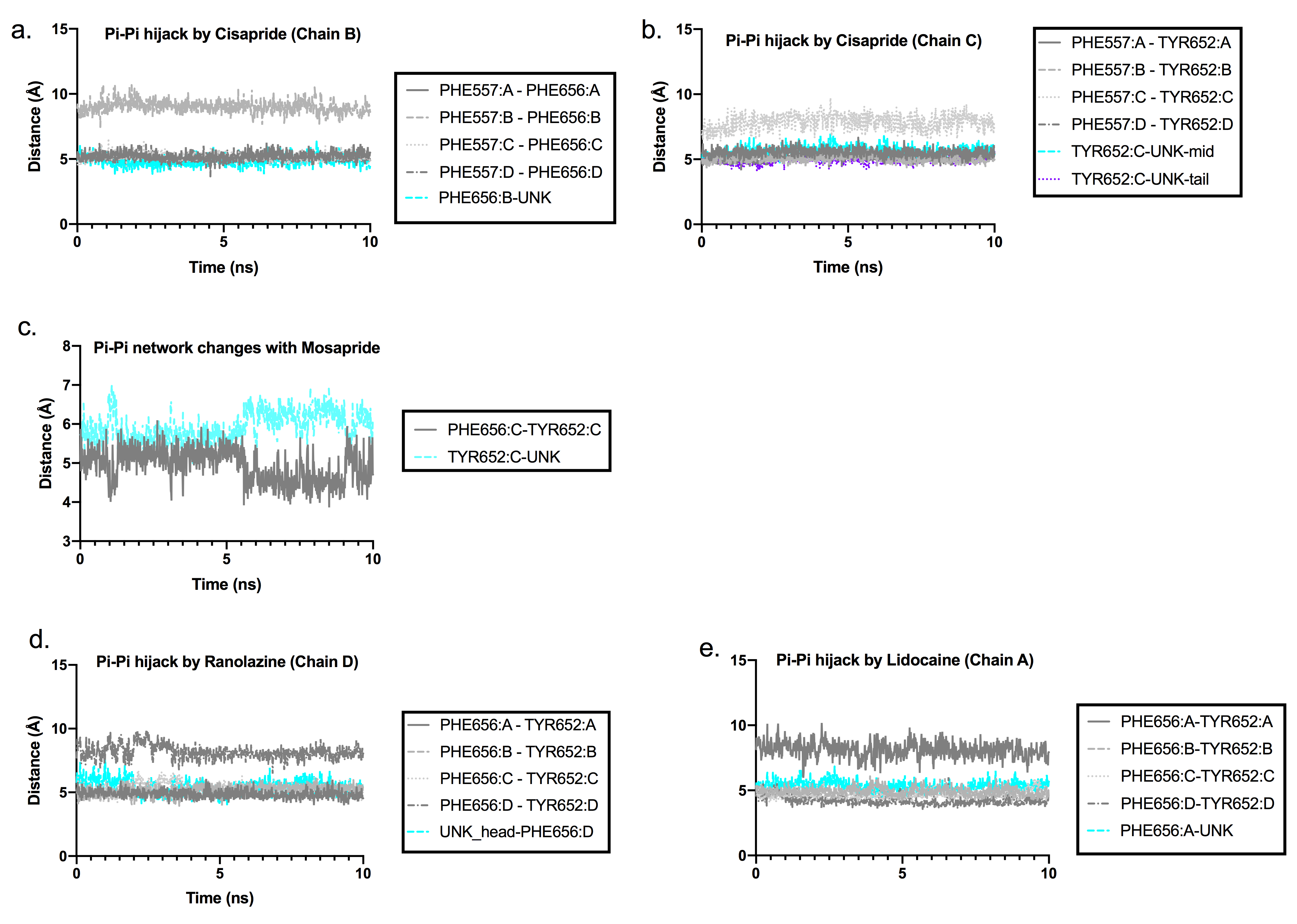
**

**Supplementary figure 21:** Distance between the drug and the aromatic residues. (a) Cisapride interactions with PHE656 residue of chain B disrupts the pi-pi interactions of PHE656:B and PHE557:B residue; (b) Cisapride interactions with TYR652 residue of chain C weakens the pi-pi interactions between TYR652 and PHE656 residues of chain C; (c) Mosapride interactions with TYR652 residue of chain C weakens the pi-pi interactions between PHE656 and TYR652 residues of chain C; (d) Ranolazine head ring interactions with PHE656 residue on chain D disrupts the pi-pi network between PHE656:D and PHE557:D; (e) Lidocaine interactions with PHE656 residue of chain A disrupts the pi-pi interactions between PHE656 and PHE557 residue of chain A.


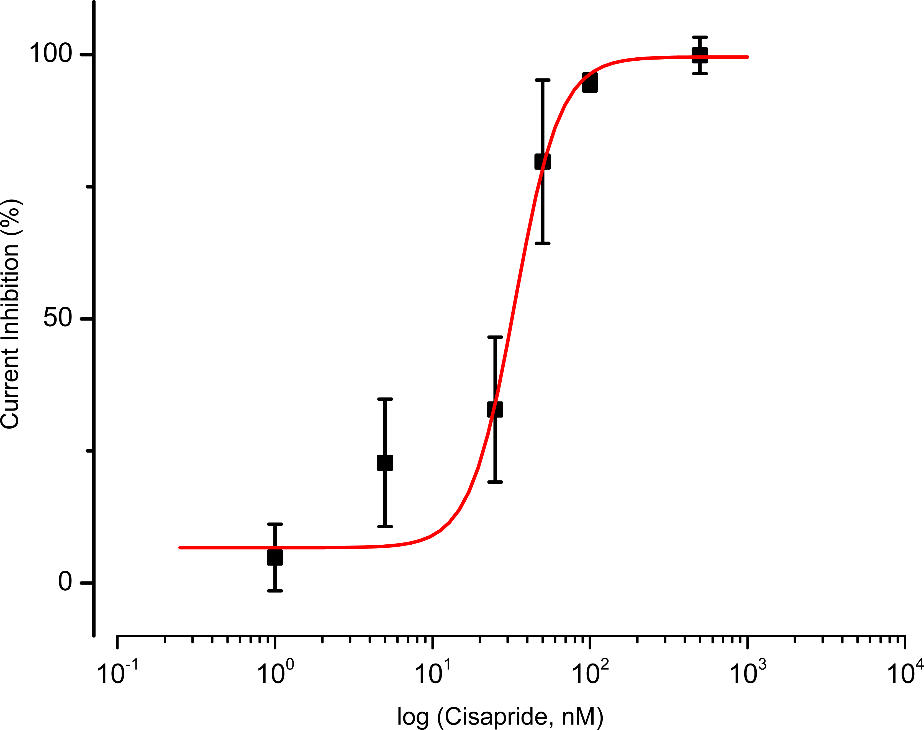


| **Supplementary Table 4**. Percent current inhibition of  WT by Cisapride for IC_50_ | | |
| --- | --- | --- |
|  |  | **hERG _WT_** |
| **Compound** | **Concentration**  **(nM)** |  |
| **Cisapride** | 1 | 4.81% ± 6.29 |
|  | 5 | 22.73% ± 12.08 |
|  | 25 | 32.82% ± 13.72 |
|  | 50 | 79.73% ± 15.46 |
|  | 100 | 94.72% ± 1.61 |
| **n =** |  | 2 to 4 |

**Supplementary Figure 22:** IC_50_ graph of hERG _WT_ with Cisapride. The IC_50_ is 33.42 ± 4.25

**Supplementary text**

**Steered molecular dynamics**

Steered molecular dynamics simulations were used to sample the starting structures for ABF windows for each pose of the drug-bound channel complexes. In order to avoid drifting movement of the protein during the pulling process, the C-alpha atom for ILE462 residues present in the S2 helix of the VSD segments in all four monomers was restrained using harmonic constraints applied to the Z direction. The choice of this residue was made such that it is far from the pulling pathway. The bound drug was pulled with a constant velocity of 0.25 Å/ps in the Z direction using a spring constant (k) value of 10 kcal/molÅ^2^. Each simulation were run for 150 ps to cover a distance of 37.5 Å, which was sufficient enough to get the drug completely out to the bulk solvent. SMD trajectory was used to sample the frames in which the drug was found in the defined window range (i.e., within the defined 2 Å window, such as -1 to -3, -3 to -5 and so on).
